# Influence of anthropogenic activities on metals, sugars and PAHs in PM_10_ in the city of Fez, Morocco: Implications on air quality

**DOI:** 10.1007/s11356-024-32740-0

**Published:** 2024-03-11

**Authors:** Nabil Deabji, Khanneh Wadinga Fomba, Eduardo José dos Santos Souza, Abdelwahid Mellouki, Hartmut Herrmann

**Affiliations:** 1 Atmospheric Chemistry Department (ACD), Leibniz Institute for Tropospheric Research (TROPOS), Permoserstraße 15, 04318 Leipzig, Germany; 2 Université Mohammed VI Polytechnique (UM6P), Lot 660 Hay Moulay Rachid, 43150 Ben Guerir, Morocco; 3 Institut de Combustion Aérothermique Réactivité Et Environnement, OSUC-CNRS, 1C Avenue de La Recherche Scientifique, 45071, CEDEX 2 Orléans, France

**Keywords:** Particulate matter, Air quality, PAHs, Sugar compounds, Trace metals, Traffic emissions, Biomass burning

## Abstract

Particulate matter (PM) is an important component in the atmosphere, affecting air quality, health, radiation balance, and global climate. To assess regional air quality in the city of Fez, an intensive field campaign was carried out in the autumn of 2019 in the Middle Atlas region of Morocco. Aerosol sampling was performed simultaneously at two urban sites in the city of Fez: (1) Fez University (FU), a sub-urban site, and (2) Fez Parc (FP), an urban site located in the city center of Fez, using PM_10_ collectors. Various laboratory analyses were carried out, including PM mass, trace metals, inorganic ions, OC/EC, sugar compounds, and PAHs. The results indicate that the PM_10_ mass (61 ng m^−3^) was comparable at both sites, with a 37–107 ng m^−3^ range. Most of the 19 investigated PAHs at the FU site (10.2 ± 6.2 ng m^−3^) were low-molecular-weight PAHs, while the most abundant PAHs at the FP site (6.9 ± 3.8 ng m^−3^) were mainly higher-molecular-weight PAHs. A diagnostic ratio analysis at both sites indicated that PAHs originated from fossil fuel combustion and traffic emissions from diesel engines, with Ant/(Ant + Phe) and Flu/(Flu + Pyr) ratios averaging 0.22 and 0.84, respectively. PMF analysis identified traffic emissions as a major source (30%), with secondary inorganic aerosols (20%) and biomass burning (14%). Polar plots highlight the dominance of local anthropogenic activities in PM pollution, with vehicular emissions, industrial activities, and biomass burning. This study shows that local sources and combustion processes significantly contribute to PM_10_ sources in Morocco, providing insights into air pollution mitigation in North Africa.

**Supplementary Information:**

The online version contains supplementary material available at 10.1007/s11356-024-32740-0.

## Introduction

Air pollution in urban areas can cause environmental health problems, leading to morbidity such as aggravated chronic heart and lung disease, especially in vulnerable populations, as well as mortality in some cases. In addition, deteriorating urban air quality increases lung cancer, stroke, heart disease, and respiratory diseases. Regarding mortality, air pollution is the most significant risk factor among all environmental pollutants, and particulate matter exposure ranked fifth among all global risk factors in 2015, contributing to 4.2 million deaths yearly (Cohen et al. 2017). Furthermore, the World Health Organization (WHO) has reported that ambient particulate matter is the primary cause of air pollution, as it affects a larger number of individuals than any other pollutant (WHO 2021).

Particulate matter (PM) consists of liquid and solid particles suspended in the air, including organic and mineral substances. These particles are important constituents of the atmosphere because of their influence on air quality, the environment, and ecosystems. Aerosol particles can originate from various sources, including local and regional emissions and long-range transported pollution. Therefore, assessing a detailed analysis of the particle composition of PM can be used to identify local and transported sources. Besides source attribution, it is also helpful to understand their impacts on human health and the environment. Particles with a diameter of less than 10 µm (PM_10_) are particularly harmful to human health. Numerous epidemiological studies have reported a causal link between exposure to these particles and mortality (Ostro et al. 2015; Turner et al. 2020; Anenberg et al. 2020; Delgado-Saborit et al. 2021; Bouma et al. 2023).

Few studies in African cities have often been focused on assessing PM_10_ levels, with results indicating annual average PM_10_ concentrations that exceed permissible WHO levels (Petkova et al. 2013; Naidja et al. 2018; Chatoutsidou et al. 2019; Chtioui et al. 2019; Agbo et al. 2021; Nducol et al. 2021; Adesina et al. 2022). Road traffic, heavy industry, and biomass burning of waste are frequently the sources of PM_10_ in urban areas (Naidja et al. 2018). Nevertheless, PM_10_ chemical characterization studies are needed to evaluate potential health risks, control emissions, and improve urban air quality. Likewise, the few studies conducted in Moroccan cities have also mainly reported PM_10_ and some major gas-phase pollutant values such as O_3_, NO_x_, and SO_2_ (Inchaouh et al. 2017; Tahri et al. 2017; Otmani et al. 2020; Bounakhla et al. 2023). Other air quality modeling studies investigated the PM_10_ variation in some urban cities in Morocco based on the inventory of local anthropogenic emissions and the application of machine learning techniques to evaluate atmospheric circulation and PM_10_ concentrations (Ajdour et al. 2020; Khomsi et al. 2020; Sekmoudi et al. 2021; Saidi et al. 2023). In some studies, gaseous pollutant levels with high ozone concentrations of up to 157 μg m^−3^ and PM_10_ levels, exceeding the WHO threshold limits, have been reported during the summer and spring at urban sites in Marrakech and Kenitra, Morocco (Inchaouh et al. 2017; Agbo et al. 2021). Measurement campaigns dedicated to both the inorganic as well as the organic chemical composition characterization are still very sparse in most African countries, including Morocco. Background aerosol composition has been studied at a remote high-altitude site in Northern Morocco (Deabji et al. 2021). This study from our group indicated that the characterization of air pollutants in this region could be influenced by several factors, including local emission sources, proximity to different source regions, as well as meteorological conditions, such as humidity, temperature, wind direction, and wind speed. Another study conducted in Morocco demonstrated that local biomass combustion contributed more to the composition of inorganic aerosols than long-distance transport (Benchrif et al. 2018). However, most of these air quality studies have mainly used monitored and modeled data without considering the assessment of chemical composition and its impact on health. Thus, studies are needed to acquire new knowledge and reduce uncertainty concerning atmospheric composition and the effect of aerosols on health in this region.

The present study aims to analyze the daily variations in PM_10_ concentrations in Fez, Morocco, while also characterizing the chemical composition of particulate matter, specifically focusing on metals and organic compounds. Moreover, the study evaluates how natural and anthropogenic activities contribute to PM_10_ budgets in the Middle Atlas region. Additionally, this study aims to examine the source apportionment of aerosol particles in Morocco using PMF as a receptor model and identify their potential spatial origins, providing insights into the air quality in the area.

## Methods

### Site description and particle sampling

An intensive campaign was conducted from September to October 2019 in Fez, an urban center in the north of Morocco. Fez is ca.180 km east of Rabat, situated between the Middle Atlas and the Rif Massif at an altitude of 414 m asl. With a population of about 1.15 million inhabitants and an area of 94 km^2^, it is the second largest city in Morocco. The intense economic activity of the city, demonstrated by heavy road traffic and many industrial activities, strongly influences the air quality of Fez. In addition, due to the proximity of this region to the Sahara Desert, which is located to the south and southeast, air quality is often affected by dust events (Deabji et al. 2021). A hot, dry summer and a cold winter are the dominant climate characteristics of the study area. Fez experiences suffocating heat and high temperatures during summer, with an average temperature of 30°C and a maximum temperature of 40°C. Unlike the cold months, the possibility of rain is almost non-existent in summer. Winters in Fez are cold with high precipitation probabilities, and minimum temperatures during the coldest months are 3°C.

The campaign was conducted from the 15th of September to the 15th of October 2019 simultaneously at two sites: (1) Fez University (FU) located at the Presidency of Sidi Mohamed Ben Abdellah University (3° 30′ 44.26′′ N, 76° 18′ 27.40′′ W; height ~ 18 m), and (2) Fez Parc (FP) an urban site situated at the Prestigia Park in Fez’s city center (3° 30′ 44.26′′ N, 76° 18′ 27.40′′ W; height ~ 5 m). The FU site in the southern part of Fez is only 450 m from a busy road (route N8), where buses, taxis, and citizens’ vehicles run on gasoline and diesel. Moreover, this road is frequently used by large trucks transporting supplies to access Fez from the city’s southern entrance. At about 50 m from the sample site is a local road (Rocade Sud), which receives many motor vehicles because of surrounding gas stations and hypermarkets. Thus, vehicle emissions are the leading local combustion sources at the FU site, while other sources of emissions are not located nearby. The FP site is located in the northern part of the city of Fez at 600 m from the Avenue des Forces Armées Royales (Route N4), one of the most frequented roads in the city. The FP site is located in the center of Fez City, which is significantly populated and surrounded by several main roads for local and national transportation. In addition, two industrial zones are located 2 km to the northeast (Quartier Industriel Doukkarat) and 4 km west (Quantier Industriel Sidi Brahim). Moreover, several construction works took place in the surroundings of the FP site at the same time as this study. The distance between the two sampling sites is about 6 km, as the Fez map indicates (Fig. S1).

A High-Volume Digitel (DHA-80) sampler was operated at a flow of 500 l min^−1^ to collect PM_10_ samples on pretreated quartz fiber filters at FU, while a low-volume sampler (LVS) was operated at the FP. A total of 120 samples were collected from the day and night (12 h) sampling routine. The collected filters were frozen at − 20°C after sampling and later transported to TROPOS (Leipzig, Germany) for chemical analysis. Vaisala WXT520 weather stations with a 1-s resolution were used to measure ambient meteorological parameters at both sites. This included ambient temperature, relative humidity, precipitation, wind speed, and direction. The inlet height was placed 3 m above the roof at FU and 7 m above ground level at FP.

### Laboratory analysis

Analysis of the chemical composition of the samples was conducted to determine the PM mass, water-soluble ions, and trace metals. In addition, the organic carbon (OC), elemental carbon (EC), and polycyclic aromatic hydrocarbon (PAH) contents were also investigated. The mass concentration of the collected filters was determined using a microbalance (Model AT261 Deltra Range, Mettler).

Carbonaceous aerosol fractions (OC & EC) were analyzed with the help of a thermo-optical instrument (Sunset Laboratory Inc. USA) using the EUSAAR2 protocol, as described by Cavalli et al. (2010). In this study, a Curie-point pyrolyzer coupled with a gas chromatography-mass spectrometry (GC–MS) system was used to detect polycyclic aromatic hydrocarbons (PAHs) (Neusüss et al. 2000; Deabji et al. 2021). Carbohydrates were quantified with high-performance anion exchange chromatography coupled with a pulsed amperometric detection instrument (Iinuma et al. 2009). The chemical characterization of organic sugar compounds involved the analysis of a total of 12 sugar compounds. The list of sugar compounds includes levoglucosan, mannosan, galactosan, mannose, glucose, galactose, fructose, sucrose, arabitol, inositol, erythritol, and mannitol. A detailed procedure for this analysis can be found elsewhere (Iinuma et al. 2009).

Trace metals were analyzed with the S2-PICOFOX total reflection X-ray fluorescence spectrometer. The samples were prepared by digesting three spots of 8-mm diameter each in 0.375 mL of hydrochloric acid and 1.125 mL of nitric acid using a microwave (MARS 6). The digested solution and an internal standard mixture of gallium and yttrium were then pipetted onto polished TXRF sample carriers, dried at ca. 80 °C, and measured with the TXRF at two different angles, 0° and 90°. Further details have been reported in the literature (Fomba et al. 2020; Deabji et al. 2021). The water-soluble ions were determined using a standard ion chromatography technique (ICS3000 Dionex) equipped with a micro-membrane removal unit and an automatic eluent system for methanesulfonic acid for anions and potassium hydroxide for cations (Fomba et al. 2013). For each sample, three spots with a diameter of 2 cm were punched out of the filter. These spots were then extracted in deionized water and shaken for two hours. A 0.45-μm unidirectional syringe filter was used to filter and eliminate any insoluble matter from the extracts that were subsequently analyzed by the IC.

### Data processing

Ninety-six–hour back trajectories were calculated using the NOAA HYSPLIT version 4 model with input data from the Global Data Assimilation System (GDAS 1°) (Stein et al. 2015). These trajectories were computed hourly at an altitude of 500 m above the model ground level. To interpret the data, we then classified the calculated back trajectories into five groups using the cluster approach (Mace et al. 2011). The mixing depth data were estimated using the HYSPLIT model. Additionally, the EC tracer approach was used to estimate the primary fraction (POC) as well as the secondary fraction (SOC) of OC (Pio et al. 2011). The minimum value of the OC/EC ratio was used to identify the primary and secondary OC fractions in aerosol components. Enrichment factors (EFs) analysis was performed based on average soil concentrations, resuspended PM_10_ concentrations, and upper crust geological concentrations (Wedepohl 1995). Moreover, the diagnostic ratios of PAHs were calculated and compared with the reported values in the literature in order to distinguish between the various sources contributing to organics (Tobiszewski and Namieśnik 2012). Pearson correlations were conducted to evaluate the presence of linear correlation across chemical species. The significance of these coefficients (*r*) was determined by assessing if the slope substantially deviated from zero (*p* value < 0.05). A Mann–Whitney *U* test, suitable for non-normal distributions, was conducted to assess the disparities across the dataset. A significance level of 0.05 was used to determine statistical significance.

Within the present study, the source apportionment of PM_10_ levels in Fez was conducted employing positive matrix factorization (PMF) using the US Environmental Protection Agency (EPA) PMF version 5.0, a receptor model (Paatero and Tapper 1994; Paatero 1999). This robust statistical approach utilizes the concentrations of various species analyzed in PM_10_ samples collected throughout the sampling period, alongside the associated measurement uncertainties as model inputs, to assign various sets of species with similar associations to a mathematical factor, which can be attributed to a source activity. The treatment of uncertainties, including those for values below the limit of detection and missing data, followed the methodology suggested by Brown et al. (2015). The critical step of determining the optimal number of factors for the PMF model incorporated quantitative and qualitative metrics. This entailed evaluating the variation of the PMF quality of fit parameter (*Q*) across a range of factors (from 2 to 8 factors) and examining the consistency of particle mass concentration profiles, temporal contribution patterns, and the correlations between modeled and measured aerosol mass concentrations. Through this comprehensive analysis, we identified seven distinct factors as the most reasonable representation of source contributions affecting PM_10_ levels in Fez, enabling a nuanced understanding of the various aerosol sources within the region.

Bivariate polar plot analyses were applied to identify source regions of PM_10_. The PMF factors were combined with polar plots, allowing for a detailed investigation into the sources and spatial distribution of pollutants. These plots divide PM_10_ concentrations into wind speed-direction sections and determine the average concentration of PMF factors for each sector (Carslaw and Beevers 2013). The R OpenAir package was used to process the meteorological data and chemical species concentrations in order to produce the bivariate polar diagrams (Carslaw and Ropkins 2012).

## Results and discussion

### Meteorology and air mass origins in Fez

The meteorology data statistics for various weather parameters in Fez are provided in Table 1. Starting with temperature (°C), the mean value of 24 °C suggests a moderately warm climate. The standard deviation of 4.8 °C indicates some variability in daily temperatures, with a minimum of 15 °C and a maximum of 36 °C, reflecting the daily range. Relative humidity averages 46%, indicating moderately dry conditions, although it can range from 13 to 89%, highlighting potential variations in water content during the sampling measurement. Solar radiation has a mean of 204 W m^−2^ but a relatively high standard deviation of 271 W m^−2^, suggesting significant fluctuations in incoming solar energy between day and night. Wind speed averages 1.5 m s^−1^, indicating generally light breezes, with a minimum of 0.1 m s^−1^ and a maximum of 5 m s^−1^. Moreover, the wind direction (degrees) predominantly aligns with an average of 193° but with a wide standard deviation of 76°, indicating changing wind patterns. The FU site is dominated by northwesterly winds with moderate wind speeds (2–4 m s^−1^). In comparison, the FP site is characterized by south-westerly winds with lower speeds (< 2 m s^−1^), as shown in Fig. S2*.* Lastly, the pressure averages at 969 mbar, with a standard deviation of 1.9, ranging from 964 to 974 mbar, suggesting relatively stable atmospheric pressure conditions. Overall, these data provide insights into the meteorological conditions experienced in Fez, highlighting the weather patterns and variations in temperature, humidity, solar radiation, wind, and pressure. Details of the temporal variation of local meteorology parameters, such as relative humidity, temperature, wind direction, and speed during the campaign, can be found in Fig. S3.

**Table 1:** Summary statistics information of meteorology data variables in Fez, including mean, standard deviation (SD), minimum (min), and maximum (max) values

Parameter	Mean	SD	Min	Max
Temperature (°C)	24	4.8	15	36
Relative humidity (%)	46	16	13	89
Solar radiation (w/m^2^)	204	271	-	882
Wind speed (m s^−1^)	1.5	0.81	0.1	5
Wind direction (degrees)	193	76	23	317
Rainfall (mm)	-	-	-	0.2
Pressure (mbar)	969	1.9	964	974

Backward trajectories of air masses help assess the impact of emissions from local sources versus emissions from regional sources over Fez. Figure S4a illustrates cluster results representing the origin and pathways of air masses reaching the urban site of Fez, providing valuable information on potential source regions and atmospheric transport patterns influencing local air quality. The study period is dominated (46% of the total trajectories) by cluster 2 when air masses move over the Atlantic Ocean and especially at low altitudes along the coasts of Portugal, Spain, and North Africa during the last 48 h before reaching Fez. Cluster 1 (35%) corresponds to slow-moving air masses from Southern Portugal at relatively low altitudes across the Atlantic Ocean and later Northern Morocco. Cluster 3 (8%) follows a similar trajectory but much faster, with clean marine air masses from the Atlantic Ocean. Cluster 5 air masses (7%) travel from the East coast of Spain, passing over the Mediterranean Sea for most of the journey. Finally, cluster 4 (4%) defines air masses with high wind speed at high altitudes from the Sahara Desert over the Atlantic Ocean.

Several factors, including meteorology and the mixing depth (MD) of air parcels, can influence air quality in Fez. Indeed, mixing depth refers to the vertical extent of the atmosphere where pollutants and other substances are mixed and dispersed. The average MD in the urban area of Fez varied considerably during the measurement campaign due to various factors such as location, meteorological conditions, and local geography, as shown in Fig. S4b. The MD varies between 100 and 800 m, with an average of 400 m. A vertical increase in boundary layer height was observed until September 25th. However, a decline in MD was observed until the end of September. In contrast, a wide variation of MD was observed from early October until mid-October, which may significantly influence urban pollutants.

### Temporal and spatial variation of PM_10_ mass

**Fig. 1:**
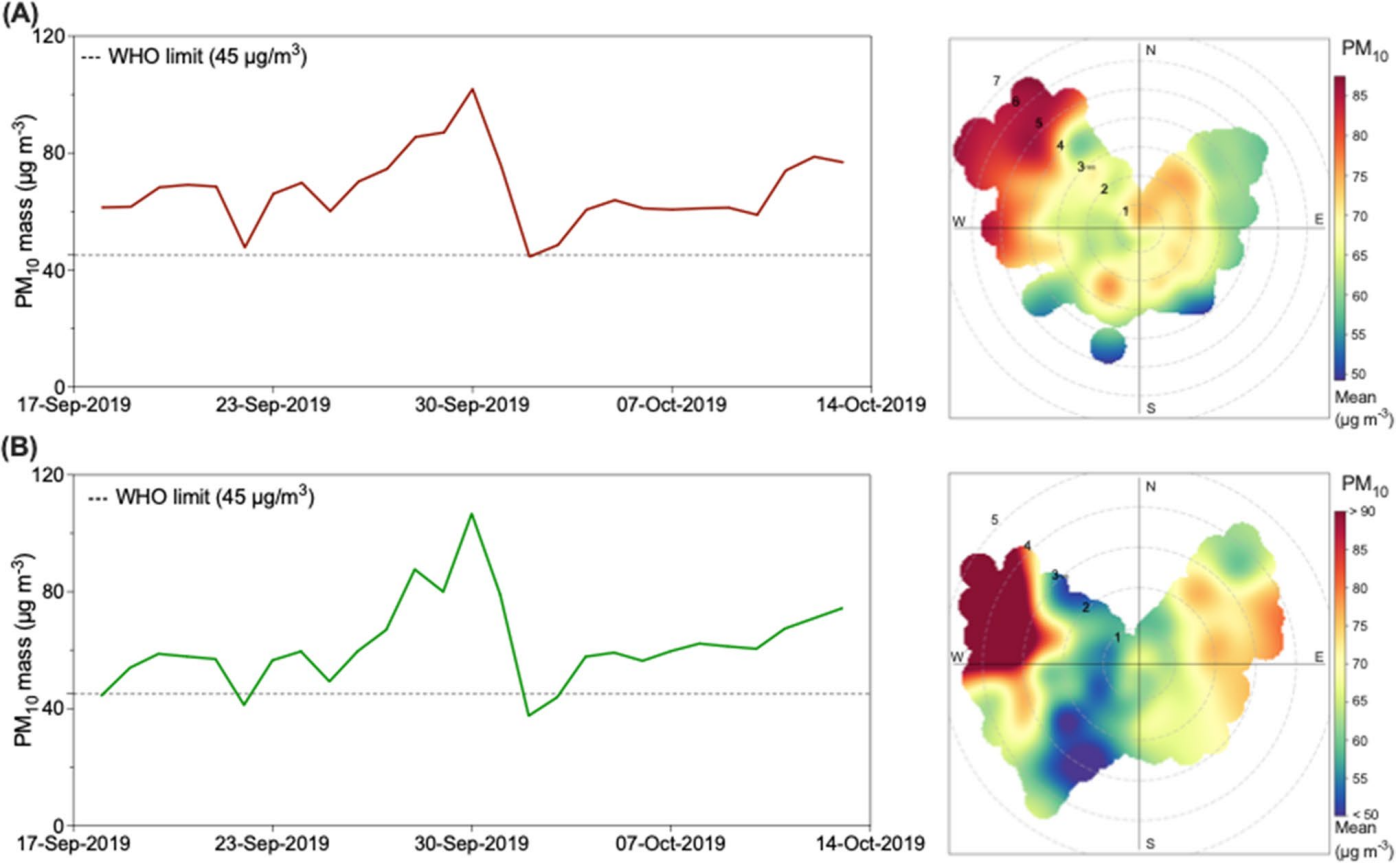
Time series of PM_10_ mass from the 15th of September to the 15th of October 2019 and polar plot of PM_10_ mass at (A) FU and (B) FP sites

The average daily PM_10_ at the FU site (67.3 μg m^−3^) was comparable with that at the FP site (61.5 μg m^−3^), as shown in Fig. 1. Initially, PM_10_ concentrations were in the range of 50–60 μg m^−3^ at the start of the campaign at both sites. However, PM_10_ concentration recorded at FU was, on average, 32% higher than at the FP site from the beginning until the end of September, despite a similar trend observed at both sites. This difference can be attributed to different local emissions combined with the high temperatures during this period. In effect, warm and dry conditions, as evidenced by the correlation of PM_10_ with temperature (*r*^2^ = 0.54; *p* < 0.05) and the inverse correlation with humidity (*r*^2^ = 0.55; *p* < 0.05), as shown in Fig. S5, facilitate the resuspension of mineral or road dust in these sites. This was observed mainly in the FU site due to its more substantial influence by car traffic. Consequently, aerosol particle mass continuously increased from September 23rd to 29th, 2019, reaching a maximum of 105 μg m^−3^. Indeed, Saharan dust transport from the south was observed during this period, starting on the 28th of September until the 1st of October, when it reached the planetary boundary layer. During the middle of the campaign, from the 2nd of October to the 5th of October 2019 the days with the lowest PM_10_ concentrations were observed, characterized by air masses from the North Atlantic Ocean that mainly were transported at high altitudes before arriving at the sites. The samples with the highest concentrations often correlated with days of higher wind speed (> 2 m s^−1^). The impact of the winds from the north on the PM was low throughout the campaign.

The 5th percentile of the PM_10_ concentrations was used as an approach to identify the reference periods representative of background conditions (Deabji et al. 2021). Accordingly, the background PM_10_ mass concentration in Fez was estimated to be 40 μg m^−3^ during the sampling period. The average PM_10_ concentration at Fez of 64 μg m^−3^ was nearly twice as high as the regional background concentration reported at the AMV (35 μg m^−m^) (Deabji et al. 2021). Moreover, approximately 81% of the samples exceeded the WHO’s recommended daily limit of 45 g m^−3^, as highlighted in Fig. 1a, b.

The spatial variation and emission sources of PM_10_ at FU and FP were characterized using the bivariate polar plot approach, as shown in Fig. 1. This approach combines PM_10_ concentration with wind direction and wind speed. Local emissions and wind direction were important factors in assessing the PM source regions in Fez. Based on the data presented in Fig. 1, the polar plots for both locations indicate two main source regions where PM_10_ concentrations are high. The first source region is connected to northwesterly winds with wind speeds greater than 3 m s^−1^, while the other region is associated with northeasterly winds with lower wind speeds below 3 m s^−1^. At the FP site, the high PM_10_ concentrations in the northeasterly direction are largely due to the industrial facilities situated in the nearby Sidi Brahim industrial area. The high PM_10_ concentrations in the northwesterly direction are often related to episodes of air pollutant transport from burning activities, which typically occur during high wind speeds and dry weather conditions across the region. However, despite the slow wind speed (less than 2 m s^−1^) during northeasterly winds observed at FU, the concentration remains relatively high in the range of 70–80 μg m^−3^, indicating a significant contribution of road traffic from the national road N°8, less than 80 m from the site. In contrast, bivariate PM_10_ plots at the FP station demonstrated that low PM events occurred at slow wind speeds (> 3 m s^−1^) in the West sector.

The average PM_10_ concentration in this study for Fez was in good agreement with studies conducted in other urban areas of Morocco and North Africa, as summarized in Table 2. However, PM_10_ levels in Fez were up to twice as high as those in Tetouan (31 μg m^−3^; Benchrif et al. 2018), and Rabat (36 μg m^−3^; Elass et al. 2020). These two sites are mainly urban background sites located on the edge of the city. Firstly, the Tetouan site shows lower values than Fez as it is strongly influenced by clean marine air masses combined with rainfall conditions. Secondly, measurements at Rabat, an urban background site, were taken in early spring, which explains the relatively lower concentrations recorded.

**Table 2:** Comparison of mean concentrations of PM_10_ in the city of Fez with those obtained from studies reported in Morocco and African cities. The concentration of PM_10_ mass is given in units of μg m^−3^. The filter approach is based on gravimetric analysis

N°	Site	Type	Period	Method	PM_10_	Reference
1	Fez, Morocco	Urban	Sep–Oct 2019	Quartz filters (12 h)	61	**This study**
2	Marrakech, Morocco	Urban	2009–2012	Real-time physical–chemical analyzers (hourly)	55	Inchaouh et al. 2017
3	Kenitra, Morocco	Urban	Jun 2007–May 2008	Polycarbonate filters (24 h/weekly)	161	Tahri et al. 2017
4	Tetouan, Morocco	Urban	May 2011–Apr 2012	Teflon filters (20 min/1 h)	31	Benchrif et al. 2018
5	Rabat, Morocco	Urban	Feb–Mar 2020	Optical measurement (15-min intervals)	36	Elass et al. 2020
6	Constantine, Algeria	Traffic	Mar 2011–Nov 2011	Quartz filters (24 h)	105	Terrouche et al. 2016
7	Sfax, Tunisia	Urban	2009–2010	Optical measurement (15-min intervals)	80	Dammak et al. 2020
8	Cairo, Egypt	Industrial	May–Oct 2010	Quartz filters (24 h)	154–360	Lowenthal et al. 2014
9	M'Bour, Senegal	Rural	2006–2007	Tapered element oscillating microbalance	108	de Longueville et al. 2013
10	Sapele, Nigeria	Industrial	May–Oct 2010	Glass fiber filters (8 h)	104–434	Ediagbonya et al. 2016

Furthermore, the geographical situation of the city of Fez, surrounded by the Atlas Mountains with low wind speed, can influence the atmospheric circulation, leading to enhanced stagnation of pollutants. Moreover, the average PM_10_ concentration here is comparable to a study conducted from 2009 to 2012 in Marrakech (55 μg m^−3^), an area with high traffic emissions with PM_10_ concentrations ranging from 50 to 110 μg m^−3^ (Inchaouh et al. 2017). Indeed, the Marrakech site has similar characteristics to Fez, as it is located in the central Haouz plain, bordered by the High Atlas Mountains to the south. Similar PM_10_ levels have been observed during summer when resuspension is responsible for a large proportion of PM_10_ emissions due to the region’s dry, windy atmosphere. In addition, the geographical location of the city of Fez, surrounded by the Atlas Mountains, can influence particle dispersion, favoring pollutant stagnation, as explained above. According to another study by Tahri et al. (2017), between June 2007 and May 2008 in Kenitra City, the average PM_10_ concentration observed (110 μg m^−3^) was much higher than in Fez due to road traffic and multiple industrial emissions. The Kenitra site is located in the northwestern part of Morocco, with a high concentration of industries such as thermal power plants, chemical, petrochemical, metallurgical, and other industries, generating different pollutants.

Although studies have been carried out in a few cities in Morocco, there is no continuous routine monitoring of particulate matter, and only a few studies have reported annual mean PM_10_ levels. In most studies, air quality monitoring remains limited to measurements of gaseous pollutants and optical measurements of PM with limited sampling times and no details of aerosol particle sources. In addition, it is essential to point out that various air quality monitoring methods were used, thereby implying difficulties in comparing data between studies. Nevertheless, PM_10_ results remained comparable with studies reported in Morocco.

When compared to measurements outside Morocco, as given in Table 2, the PM_10_ concentrations in Fez were relatively low compared to other North African city’s levels of pollution, such as Cairo (154–360 μg m^−3^; Lowenthal et al. 2014), and Constantine (105 μg m^−3^; Terrouche et al. 2016), and Sfax (80 μg m^−3^; Dammak et al. 2020). These sites are heavily impacted by biomass burning, traffic, and industrial emissions. In Fez, high concentrations of PM_10_ were observed during the summer and early autumn, primarily due to the resuspension of soil dust and Saharan dust intrusions. However, in Sfax, Tunisia, higher PM_10_ concentrations were observed during winter-spring due to Saharan wind circulation (Dammak et al. 2020). Additionally, higher PM_10_ concentrations were observed in autumn in Fez compared to summer due to heavy traffic and industrial activities in the area, such as lead smelters and metal production (Lowenthal et al. 2014). In comparison to the sub-Saharan region, PM_10_ concentrations in Fez were significantly lower. For example, De Longueville et al. (2013) reported average annual PM_10_ concentrations of 108 μg m^−3^ in M’bour, Senegal, due to the influence of Saharan dust. Similarly, a study conducted in Sapele, Nigeria, from May to October 2010 reported average concentrations ranging from 156 to 850 μg m^−3^, up to 8 times higher than in Fez (Ediagbonya et al. 2016). Local meteorology, particularly relative humidity and temperature, greatly influenced the concentration of PM_10_ in the Fez region (Ediagbonya et al. 2016).

### Chemical composition of PM_10_

#### Main compounds in Fez PM

Table 3 provides a summary of the chemical composition of PM_10,_ including mass, OC/EC, major inorganic elements, and inorganic secondary aerosol (SIA). The OC concentration was slightly higher at the FP site (7.2 μg m^−3^) than at the FU site (6.7 μg m^−3^), while the EC concentration was slightly higher at the FU site (3.0 μg m^−3^) than FP (2.6 μg m^−3^). The concentrations of OC and EC of PM_10_ in the present study were 6 times and 12 times higher, respectively than in the isolated AMV background site (Deabji et al. 2021). The source of carbonaceous aerosols is mainly fossil fuel combustion, as indicated by the OC/EC ratio observed at FU (3.3) and FP (2.6) (Cao et al. 2011). Nevertheless, biomass burning may also influence elevated OC and EC concentrations in PM_10_. Pio et al. (2011) reported a high OC/EC ratio in biomass burning emissions during certain seasons and in rural and residential areas, which could also be the case at Fez. The OC/EC ratio was calculated to evaluate the contribution of POC and SOC emissions at each site. As a result, the estimated POC concentration at FU was 4.5 ± 1.9 μg m^−m^, and the SOC concentration was 2.2 ± 1.2 μg m^−3^, which represented 65% and 35% of PM_10_, respectively. Consequently, a positive linear trend (*r*^2^ = 0.66; *p* < 0.05) is observed between OC and EC for the urban FU site, especially when OC concentrations are above 3.5 μg m^−3^, indicating their common source, presumably from vehicular emissions (Salma et al. 2004), as shown in Fig. S6. At the FP site, there was a comparatively higher concentration of SOC (3.8 ± 1.5 μg m^−3^), while the POC concentration (3.5 ± 1.9 μg m^−3^) showed a lower contribution most likely attributed to local emission sources. The central location of the FP site may result in higher VOC emissions, leading to the formation of SOA. Additionally, the urban setting of the site could contribute to SOA production due to higher levels of anthropogenic precursors like NO_x_ and O_3_ from city traffic and industrial activities. These factors combine to create an increased SOA formation and a distinct SOC profile at FP compared to the FU site. In addition, the strong correlation (*r*^2^ = 0.90; *p* < 0.05) between SO_4_^2−^ and NH_4_^+^ in aerosol particles observed at both sites indicates the strong secondary particle formation of ammonium sulfate in Fez (Fig. S7). Consequently, SO_4_^2−^ was mainly present in the form of ammonium sulfate, probably due to the higher SO_2_ levels and higher photochemical reaction rate during this period. Conversely, NO_3_^−^ and NH_4_^+^ correlations were lower at FU (*r*^2^ = 0.58; *p* < 0.05) and FP (*r*^2^ = 0.52; *p* < 0.05) during the measurement campaign (Fig. S7). This low correlation may be attributed to temperature’s influence on the nitrogen species’ gas-particle distribution. Benchrif et al. (2018) reported a more or less similar concentration of OC (6.1 μg m^−3^) and EC (3.2 μg m^−3^) in PM_10_ in Tetouan, Morocco. Their study analyzed the chemical compositions of aerosols between May 2011 and April 2012, suggesting that carbonaceous aerosols were mainly related to local emissions by traffic than transported over long distances. Khedidji et al. (2020) reported lower contributions of OC (3.1 μg m^−3^) and EC (1.2 μg m^−3^) from September 2011 to January 2012 in Bou Ismaïl, Algeria, which is located in the south Mediterranean coastal area. Similarly to Fez, OC (3.72 μg m^−3^) and EC (1.36 μg m^−3^) mean concentrations were also significantly elevated in October (Khedidji et al. 2020).

**Table 3:** Mean with standard deviation (SD), minimum, and maximum of PM_10_ chemical components in Fez. The concentration of particle mass and major chemical components are given in units of μg m^−3^

Chemical component	Fez Uni (FU)			Fez Parc (FP)		
	Mean ± SD	Min	Max	Mean ± SD	Min	Max
Mass	67 ± 16	44	104	61 ± 23	38	107
OC	6.7 ± 1.9	1.4	10	7.2 ± 2.3	2.5	12
EC	3.0 ± 1.2	0.33	6.4	2.6 ± 1.5	0.52	6.6
POC	4.5 ± 1.9	0.50	9.8	3.5 ± 1.9	0.69	8.9
SOC	2.2 ± 1.2	0.01	6.1	3.8 ± 1.5	1.70	9.0
NO_3_^−^	2.8 ± 1.1	0.68	6.1	2.9 ± 1.0	0.63	5.6
SO_4_^2−^	2.5 ± 1.3	0.64	6.4	2.5 ± 1.1	0.76	6.1
NH_4_^+^	0.66 ± 0.42	0.06	2.0	0.71 ± 0.36	0.15	1.7
Na^+^	0.66 ± 2.26	0.16	1.1	0.77 ± 0.28	0.23	1.3
Cl^−^	0.36 ± 0.27	0.09	1.4	0.44 ± 0.32	0.12	1.4
Mg^2+^	0.17 ± 0.03	0.09	0.26	0.15 ± 0.03	0.06	0.22
C_2_O_4_^2−^	0.22 ± 0.06	0.05	0.39	0.18 ± 0.04	0.04	0.27

Quantifying OC and EC fractions provides insights into the sources and formation processes of aerosol particles (Monteiro dos Santos et al. 2016). In our analysis, aerosol carbon content on quartz-fiber filters is thermally analyzed in two phases using specific temperatures. Initially, in a helium atmosphere, organic carbon fractions (OC1–OC4) evolve at 200 °C, 300 °C, 450 °C, and 650 °C, respectively. Subsequently, in a 2% oxygen/helium mix, elemental carbon fractions (EC1–EC4) are determined at 500 °C, 550 °C, 700 °C, and 850 °C. This process identifies nine key carbon fractions: four OC, four EC, and pyrolyzed organic carbon (PC) (Pereira et al. 2023).

Figure S8 presents an analysis of OC and EC fractions in PM_10_ at two sites, FU and FP, revealing patterns in their source contributions. Notably, OC3 and OC4 are major components at both sites, comprising 23% and 31% of the total OC, suggesting a significant influence from combustion processes such as vehicle emissions and biomass burning, which are known to produce higher molecular weight OC fractions (Zhang et al. 2021). Conversely, OC1 and OC2, accounting for 12% and 14%, respectively, indicate contributions from biogenic sources or low-temperature combustion processes. Elemental carbon fractions differ between the sites. EC1 is more prominent at FP (37%) than at FU (33%), reflecting direct particle emissions from traffic or industrial sources (Gu et al. 2010). EC4’s lower abundance (6%) at both sites suggests less contribution from high-temperature combustion processes.

The concentration of SO_4_^2−^ at FU (2.6 μg m^−3^) varied from 0.64 to 6.4 μg m^−3^, while the mass concentration of NO_3_^−^ varied from 0.68 μg m^−3^ to 6.1 m^−3^ with a mean value of 2.6 μg m^−3^ (Table 3). The concentration of SO_4_^2−^ in PM_10_ was higher during the dust storm. Similarly, NO_3_^−^ followed the same pattern, indicating that the Sahara dust acts as a surface that enhances the circulation of sulfate and nitrate-related pollutants in Fez. The minimum concentrations for SO_4_^2−^ and NO_3_^−^ were observed during precipitation events. Comparing the concentrations of SO_4_^2^ and NO_3_ with the more isolated mountain AMV site, we find that the concentrations are twice as high in Fez. Other studies in Morocco show similar values for SO_4_^2−^ (2.7 μg m^−3^), while NO_3_^−^ (1.2 μg m^−3^) was found to be higher in Fez (Benchrif et al. 2018). Furthermore, NH_4_^+^, Na^+^, Cl^−^, Mg^2+^, and oxalate concentrations were comparable at both sites, suggesting that local emissions have little influence.

#### Trace metals

Table 4 presents the summary of the average trace metal concentrations and standard deviation for nineteen elements (Ca, Fe, K, Pb, Mn, Sr, Ni, Cu, Zn, V, Sb, Ti, Cr, Ba, Br, Co, Bi, Se, As). High concentrations were obtained for Ca, Fe Mn, and Ti, mainly of crustal origin. Fe (488 ng m^−3^) and K (329 ng m^−3^) recorded lower concentrations than Ca, which had the highest concentration (1866 ng m^−3^). These elements were up to twice as high at the FU site than at the FP site due to the resuspension of dust by traffic, which occurs repeatedly in cities.

**Table 4:** Mean and standard deviation (SD) of PM_10_ elemental components at Fez; concentrations are given in units of ng m^−3^

Trace metals	FP		FU	
	Mean	SD	Mean	SD
Ca	1866	1293	2712	1672
Fe	488	328	662	413
K	329	149	487	341
Zn	156	157	232	143
V	111	79	144	114
Pb	36	58	40	40
Mn	29	19	38	23
Sr	40	14	33	11
Ni	96	63	24	27
Cu	27	168	23	11
Sb	71	93	144	124
Ti	63	45	140	101
Cr	35	47	12	6
Ba	28	35	68	62
Br	43	28	44	26
Co	31	30	32	34
Bi	5.5	11	7.6	9.6
Se	11	8.4	7.3	7.2
As	0.03	0.16	0.13	0.51

When associated with other elements, the analyzed metals indicate the presence of anthropogenic sources such as construction or non-exhaust traffic emissions (Fomba et al. 2018). Nevertheless, Fe could be partly due to brake wear and muffler removal. Indeed, on the one hand, studies have reported that the association of Fe with Zn is related to fossil fuel combustion and waste incineration sources (Gao et al. 2002; López et al. 2005). On the other hand, friction and heating of tires may result in the release of zinc, which can also be one of the reliable tracers of emissions from unleaded fuel and diesel-powered vehicles (Monaci et al. 2000; Ramadan et al. 2000). Nevertheless, Fe and Zn show high correlations with mineral dust tracers like Ca, Ti, K, and Mn, suggesting that they may have originated from the same source of road dust resuspension. As a result of the combined contribution of these sources, most likely from industry emissions, the average Zn value at FU and FP reached a value of 232 ng m^−3^. Other elements, such as Pb, Cr, Cu, and Ni, are more abundant at the FP site than at the FU site. These elements could possibly originate from industrial activities such as metallurgical processes (Ni and Cu) in Sidi Brahim Industrial Area, battery manufacturing and recycling activities (Pb) in the Ain Nokbi Industrial Area, and traditional tanning processes (Cr) in the Old Medina. Although anthropogenic metal concentrations do not exceed the air quality guidelines, they remain significant (WHO 2000). The V concentration of 111 ng m^−3^ was observed at the FP site and 144 ng m^−3^ at the FU site, which is most likely associated with oil combustion. Although V may originate from ship emissions, no correlation was found with sea salt compounds such as Na^+^ and Cl^−^ at both sites, suggesting that most V-containing particles were primary particles emitted by dust and industrial emissions. However, a correlation was found with Cr, Mn, and Fe. The concentrations of Pb differed slightly between the two sampling sites. However, the mean Pb recorded at the FU site (40 μg m^−3^) exhibited significantly higher levels compared to the FP site (36 μg m^−3^). This discrepancy suggests the presence of localized lead sources near FU, potentially associated with proximity to heavy traffic areas. The levels of Pb were relatively low at both sites due to the decreased use of Pb-containing anticaking gasoline additives called leaded gasoline in Morocco. However, the observed mean Pb level was significantly lower than the WHO guideline value of 0.5 μg m^−3^ at both sites. Copper (Cu), which is characterized by significant toxicity, showed an average concentration of 27 ng m^−3^ at the FP. Nevertheless, Cu emissions can be associated with industrial areas located in the northeast and northwest, diesel engines, and the port of station wagons (Patel et al. 2019). Ni, which was up to four times higher at the FP site (96 ng m^−3^), is primarily associated with fossil fuel use, petroleum combustion, and emissions from stationary and industrial sources. However, brake wear and vehicle exhaust could also contribute to Ni in ambient air (Adamiec et al. 2016). These results indicate that the accumulation of anthropogenic metals from vehicle and industrial emissions significantly affects air quality in Fez.

A comparative study between the results obtained for trace metals in the city of Fez and those obtained in studies reported in the literature in other cities in Morocco and North Africa is presented in Table 5. Similar Cu, K, and Mn values were found in Meknes using the TXRF technique, where the particles were collected from March 2007 to April 2008 (Bouh et al. 2022). However, higher levels for Fe, Ca, and Pb were observed in that cited study because of the site’s location in the city center of Meknes near the highways where the automobile traffic is very important and not far from the railway traffic. Likewise, similar results were reported for Ca from March to May 2010 in Constantine, Algeria (Terrouche et al. 2015). The presence of Ca was mainly related to the resuspension of dust. Nevertheless, the concentrations of Ca (2712 ng m^−3^) and Fe (662 ng m^−3^) are predominantly lower than those found in other cities in Tunisia (Ellouz et al. 2013; Bahloul et al. 2015). This is because these cities could be more exposed to heavy traffic, increasing the resuspension of road-associated dust. In addition to heavy traffic, seasonal weather patterns and desert dust also contribute to PM_10_ concentrations. Specifically, prolonged anticyclonic or cyclonic situations can elevate crustal element concentrations in PM_10_. Moreover, the contribution of African desert dust, influenced by regional wind patterns and seasonal dust outbreaks, plays a vital role in the composition and levels of PM_10_. This is particularly evident in areas prone to dust transport from nearby desert regions such as Tunisia and Algeria (Terrouche et al. 2015; Bahloul et al. 2015).

**Table 5:** Comparison of trace metal concentrations in PM_10_ in the city of Fez with those reported in Morocco and North Africa. Concentrations are given in units of ng m^−3^

N°	Sampling site	Type	Ca	Fe	Cu	K	Cr	Mn	Pb	Zn	References
1	Fez, Morocco	Urban	2712	662	23	487	12	38	40	232	**This study**
2	AMV, Morocco	Remote	649	486	1.2	174	4.3	12	4.8	8.6	Deabji et al. 2021
3	Meknes, Morocco	Urban	4490	1186	33	488	92	49	154	100	Bouh et al. 2022
4	Algiers, Algeria	Urban	-	640	103	-	-	58	299	-	Talbi et al. 2018
5	Constantine, Algeria	Urban	3010	-	450	4080	-	-	900	1290	Terrouche et al. 2015
6	Sfax, Tunisia	Urban	9880	1900	-	-	-	40	130	90	Bahloul et al. 2015
7	Bouhmel, Tunisia	Urban	8220	2820	-	-	-	510	-	-	Ellouz et al. 2013

It should be noted that the concentrations of all trace metals were significantly higher than those observed at the remote high-altitude site AMV (2100 m). These results corroborate that the location of Fez City between the Middle Atlas Mountains favors the stagnation of PM and the accumulation of anthropogenic metals compared to high-altitude sites. Heavy metal levels were also lower than those found in other cities in Northern Africa in moderately polluted areas. Some exceptions were observed in Fez for Zn, which had a higher concentration than Meknes and Sfax (Bahloul et al. 2015; Bouh et al. 2022). The study conducted in Sfax previously reported that heavy metals were mainly emitted by the lead smelting industries (Bahloul et al. 2015).

#### PAHs pattern and toxicity in Fez

**Fig. 2:**
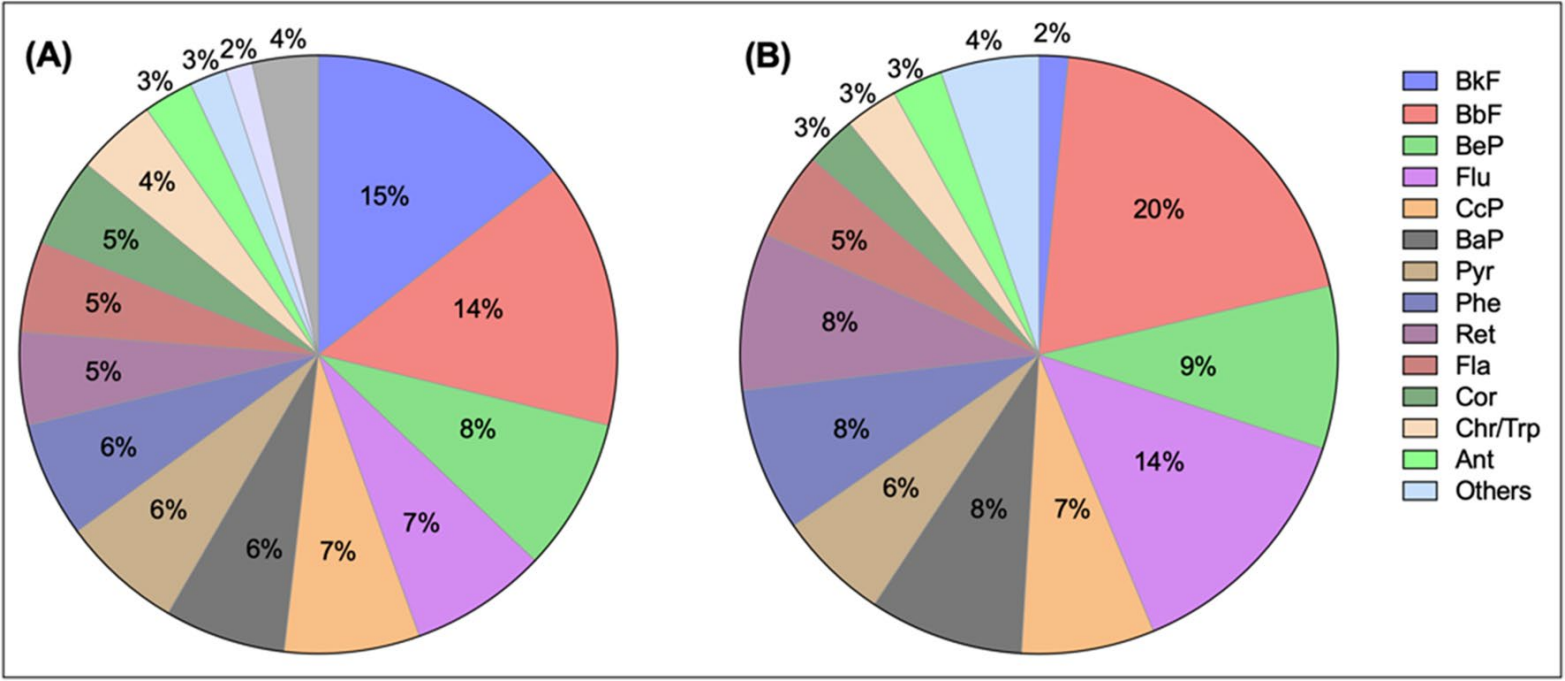
The relative abundance of PAHs measured in PM_10_ samples collected at **A** Fez University (FU) and **B** Fez Parc (FP)

Among the collected samples, 19 PAHs were quantified, including those listed by WHO and the US Environmental Protection Agency (USEPA) as potential toxic pollutants such as indeno(1–2, 3 cd)pyrene (IcdP), benzo(a)anthracene (BaA), benzo (a)pyrene (BaP), and benzo(e)pyrene (BeP) (Yang et al. 2012a; Zhao et al. 2014). The list of organic compounds with their respective symbols is shown in Table S1. The total PAH concentration was 10.2 ± 6.2 ng m^−3^ at the FU site and 6.9 ± 3.8 ng m^−3^ at the FP site. Figure 2 shows the distribution of organic compounds at each site during the sampling period. The FU site is distinguished by a dominance of five-ring PAHs such as benzo(k)fluoranthene (BkF; 15%), benzo (b)fluoranthene (BbF; 14%), BeP (8%), and six-ring PAHs including benzo(ghi)perylene (BghiP; 8%) and IcdP (7%), which accounted for about 53% of the total PAH concentration (Fig. 2). The most abundant PAHs at FP were mainly five-ring PAHs including benzo (b)fluoranthene (BbF; 20%), retene (Ret; 9%), BeP (9%), and BaP (8%). In addition, fluorene (Fla; 14%), a four-ring PAH, was vastly abundant at FP, unlike the six-ring group, which was the least abundant. Among the most abundant PAHs at the FP site, retene constituted 9% of the total PAHs. While retene is typically associated with biomass burning, it is also identified in tire-related emissions, as suggested by a recent study (Pereira et al. 2023). The presence of retene in our urban study site could, therefore, be indicative of both sources, including biomass combustion in residential heating or cooking and tire wear from the heavy vehicular traffic characteristic of urban areas. It demonstrates that each site is characterized by different source emissions of PAHs distinguished by their structure and size. PAHs are often linked to different types of combustion. Those with two or three aromatic rings, which are considered PAHs with low molecular weight (LMW), are usually associated with fuel oil and biomass burning. Coal combustion and vehicle emissions commonly contain PAHs with high molecular weight (HMW), as well as those with five or six rings. Therefore, the prevalence of HMW PAHs at the FP site is indicative of multiple sources. While this can be attributed to fossil fuel combustion, it is important to recognize that vehicular emissions, especially from light-duty vehicles (LDVs) powered by gasoline, are also a significant source of these compounds (Perrone et al. 2014). In contrast, the presence of LMW PAHs at the FU site suggests contributions from vehicular emissions. In multiple studies, chemicals like BbF, BkF, BaP, and IcdP have been identified as markers of diesel combustion (Khalili et al. 1995; Ravindra et al. 2008; Galarneau 2008; Wang et al. 2010; Ma et al. 2011; Jamhari et al. 2014). It corroborates the influence of diesel fuel from trucks and public transport at the FU site.

PAH levels at FU (10.2 ng m^−3^) and FP (6.9 ng m^−3^) are comparable to those measured in PM_10_ reported in the literature in the North African region such as (9.4–44.8 ng m^−3^; Bizerte, Tunisia), (0.4–11.2 ng m^−3^; Algiers, Algeria), (0.5–17.8 ng m^−3^; Bizerte, Tunisia), and (2.44–35.3 ng m^−3^; Bou Ismaïl, Algeria) (Ben Hassine et al. 2014; Khedidji et al. 2017, 2020; Barhoumi et al. 2018). A previous study in Alexandria City, Egypt, conducted between January 2015 and February 2016 on PM_10_ samples, showed higher concentrations of BkF, followed by BaP and IcdP. These HMW compounds are carcinogenic with high exposure in areas of traffic and vehicle emissions (Haiba and Hassan 2018). In contrast, PAH concentrations in this research were significantly higher (1.6–199 ng m^−3^) than those of PM_10_ reported in our study. Another study by Khedidji et al. (2020) reported high levels of other PAHs in Bou Ismaïl, Algeria, such as pristane and phytane, which were lower in Fez. Petroleum combustion residues, especially from traffic emissions, are often responsible for these pollutants. With respect to the PAHs, the most abundant compounds were pyrene (Pyr), fluoranthene (Fla), BghiP, BbF, Chr, and BaP. In contrast, some studies reported in an urban area of Bizerte (Tunisia) in 2009–2010 that the most abundant compounds were Pyr, Fla, BghiP, BbF, Chr, and BaP (Ben Hassine et al. 2014). The samples involved in their study were collected. The site is relatively densely populated and highly influenced by high traffic density, which could have contributed to the PAH concentrations. However, PAH measured in Fez remains significantly higher than in other studies in regions exposed to high vehicular exhaust emissions, such as the Bouira province located in Algeria (0.84–10.8 ng m^−3^) (Ladji et al. 2014). Although the concentrations were low compared to Fez, the PAHs reported were strongly associated with ultrafine particles, which can easily enter the lung alveoli and affect human health.

At both sites in Fez, the carcinogenic species BaA, BbF, BkF, BaP, and BeP were detected in the majority of PM_10_ samples. BaP is a reference for PAH carcinogenicity used to measure PAH exposure (Tilton et al. 2015). Exposure to BaP can cause short-term health effects such as skin rash and eye irritation, while long-term exposure can be fatal as BaP is a probable cancer-causing agent (Carreras et al. 2013; Khedidji et al. 2017). Consequently, the BaP values show a strong variation at both sites, as shown in Fig. S9. The average carcinogenicity level at Fez during the campaign was comparable at both sites, as shown by BaP mean values at FU (0.65 ng m^−3^) and FP (0.59 ng m^−3^). At the FU site, BaP concentrations were about twice as high during the daytime than at night, with a maximum of 2.8 ng, m^−3^ likely due to traffic-related sources, particularly fossil fuel combustion, such as gasoline and diesel. Furthermore, higher exhaust fumes from cars, trucks, motorcycles, and other motor vehicles, which contribute to increased BaP concentration, distinguish the FU site. On the other hand, BaP concentrations at the FP site were higher at night, reaching a maximum of 3.1 ng m^−3^, primarily because of residential heating and cooking activities. The incomplete combustion of solid fuels like coal, wood, and biomass in residential heating systems and traditional cooking practices contributes significantly to the emission of BaP (Sun et al. 2021). Approximately 22% of the samples collected at the FU site and 11% at the FP site exceeded the World Health Organization’s maximum allowable concentration threshold of 1 ng m^−3^. These findings highlight the potential health risks in the region.

#### Sugar compounds

The combustion of fossil fuels, industrial activities, and urbanization contribute to the emission of various pollutants into the atmosphere, including sugar compounds. The most common are anhydrosugars, monosaccharides, and sugar alcohols, commonly used as tracers of organic pollution in urban aerosols. The main tracers of biomass combustion emissions are anhydrosugars, such as mannosan, galactosan, and levoglucosan (Simoneit 2002; Marynowski and Simoneit 2022).

**Fig. 3:**
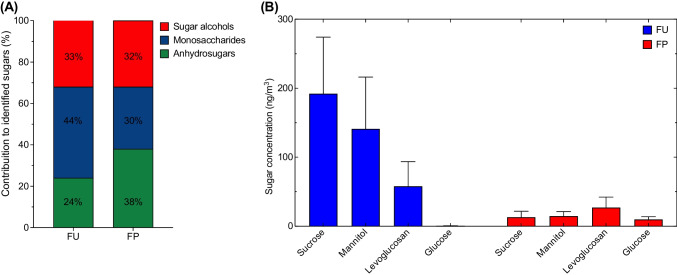
**A** Contribution of sugar groups to total identified sugars at FU and FP sites; **B** Main sugar compounds measured in PM_10_ samples at each site

The contribution of sugar groups was dominated by Monosaccharides (up to 44%) at the FU site with 221 ± 89 ng m^−3^. Nevertheless, the concentration of anhydrosugars and sugar alcohols remained significantly high at 119 ± 73 ng m^−3^ and 164 ± 77 ng m^−3^, respectively. Sugar compounds such as sucrose 192 ± 89 ng m^−3^ and mannitol 141 ± 75 ng m^−3^ dominate the sugar composition, as illustrated in Fig. 3. Nevertheless, the concentration of levoglucosan remains high, with an average of 58 ± 35 ng m^−3^ and observed peaks of up to 213 ng m^−3^ (Fig. S9). Correlation analysis reveals that sucrose correlated strongly with levoglucosan (*r*^2^ = 0.97; *p* < 0.05) and galactosan (*r*^2^ = 0.97; *p* < 0.05), suggesting a common source linked to burnt biomass emissions in Fez (Fig. S11). However, a weak correlation was found between alcohol sugars such as mannitol and primary sugars, including mannose (*r*^2^ = 0.36) and fructose (*r*^2^ = 0.26), indicating that fungal spore-related origins do not play a significant role. In addition, the average glucose concentration was low (0.27 ± 0.38 ng m^−3^), suggesting that combustion processes were not the primary source of glucose at the FU site.

In contrast, sugar concentrations at the FP site were up to 7 times lower than at FU. The sugar trend is dominated by anhydrosugars (up to 38%), mainly with a significant contribution from levoglucosan (27 ± 17 ng m^−3^), with a maximum concentration of 86 ng m^−3^. Despite the relatively high contribution of monosaccharides (30%) and sugar alcohols (32%) to total sugars, the sugar concentration of sucrose (12 ± 8.8 ng m^−3^) and mannitol (14.6 ± 6.5 ng m^−3^) remains relatively low. On a different note, FP is characterized by high concentrations of alcohol sugars such as arabitol (9.9 ± 4.6 ng m^−3^) and glucose (9.8 ± 3.9 ng m^−3^), as shown in Table S2. On the one hand, sugar alcohols show a high correlation, indicating similar sources, such as aerial pollen of plant origin. On the other hand, weak correlations were found between levoglucosan and other alcohol and primary sugars. Consequently, a similar temporal trend for glucose and sucrose indicates that they have a common source, namely pollen found in the park (Fig. 3). Indeed, glucose can be derived from leaf fragments and pollen species (Fu et al. 2012). However, sucrose correlates poorly with levoglucosan (*r*^2^ = 0.07) at the FP site, indicating they come from two different sources. In summary, emissions from biomass combustion appear to be an important source of sugar compounds at the FU site, while pollen and fungal spores play a more critical role at the FP site.

Comparing atmospheric abundances of sugar compounds in Fez with literature data is challenging, given that only a few studies in the literature have quantified sugar compounds in Africa. Nevertheless, some studies have succeeded in measuring a few compounds, mainly levoglucosan and mannitol. Levoglucosan concentrations in Fez (58 ± 35 ng m^−3^) are significantly higher than at the AMV’s remote research site in the Middle-Altas mountains (2.0 ± 1.1 ng m^−3^), showing the local contribution of biomass and combustion emissions (Deabji et al. 2021). However, in North Africa, levoglucosan concentrations (9.9–213 ng m^−3^) are comparable with the study carried out at the Southern Mediterranean Coast Zone located at Bou Ismaïl, Algeria, between September 2011 and January 2012, which reported a concentration in the range 3.8–211 ng m^−3^ (Khedidji et al. 2020). Nevertheless, levoglucosan levels in Fez are lower than those observed at the rural site in Tanzania, located in West Africa, during the dry season (99–659 ng m^−3^) (Mkoma et al. 2013). Similarly, Fez is characterized by lower ranges of Mannosan concentrations (1.5–7.9 ng m^−3^) compared to the rural site in Tanzania (10–59 ng m^−3^). This is due to the overwhelming contribution of anhydrosugars associated with emissions from mixed biomass and biofuel combustion activities (Mkoma et al. 2013).

### Enrichment factor analysis

The enrichment factor (EF) is an approach to identify metals from natural sources and those from anthropogenic activities (Zhang et al. 2008). To identify which trace elements are enriched in aerosols compared to soils, it is necessary to compare the composition of PM_10_ with the average composition of soils. This approach indicates the contribution of anthropogenic emissions on atmospheric elemental levels. Studies have used Al, Si, Ti, Fe, as well as Mn as reference elements for the EF calculation due to their abundance in the crust and less significant influence by pollution (Teixeira et al. 2009; Fabretti et al. 2009; Sakata and Asakura 2011). Although the enrichment factor approach offers a valuable tool for identifying potential pollution sources, it is subject to limitations due to variations in background concentrations, the choice of reference elements, and the assumption of a constant EF for each source category, which may affect the reliability of the results. In our study, Ti was used as a reference element, and the upper continental crust, according to Wedepohl (1995), was used. The EF of any trace element Z relative to a reference material is defined as follows:

$$EF(Z)=\frac{\left(\frac{Z}{Ti}\right){\text{Aerosol}}}{\left(\frac{Z}{Ti}\right){\text{Soil}}}$$

*Z* and *Ti* are the concentrations (ng m^−3^) of the investigated element and the reference compartment, respectively.

**Fig. 4:**
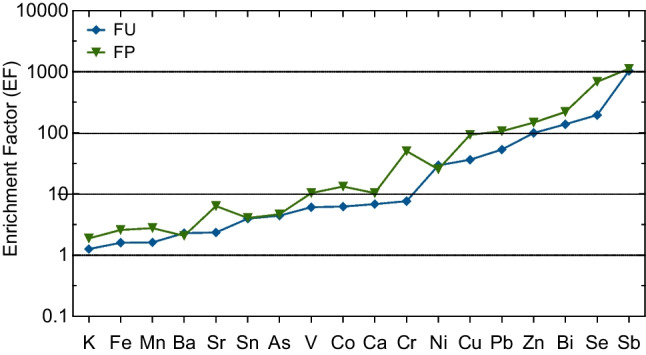
Crustal enrichment factor (EF) of trace metal at FU and FP sites

Figure 4 illustrates the calculated EF values of K, As, Fe, Mn, Ba, Sr, Sn, V, Co, Ca, Cr, Ni, Cu, Pb, Zn, Bi, Se, and Ba at both sites in Fez city. The EF increases with the contribution of anthropogenic origins according to the degree of enrichment. Thus, the elements studied were assembled into three groups: a first group where values below 10 included elements of crustal origin with minimal enrichment. A second group of EFs in the range of 10–100 contains moderate to significant enriched elements. Finally, a third group of EFs (> 100) contains elements of very high enrichment. The results showed the EF values of K, As, Fe, Mn, Ba, Sr, Sn, V, Co, and Ca were all less than 10, indicating that natural sources dominated the presence of these elements in airborne dust. Except for As, EFs were 1.5 to 3 times higher at the FP site than at the FU site. Indeed, the EF for As at FP was less than 10 due to the low activity of the smelting furnace (Dai et al. 2015). The EF for K relative to the soil concentration was also low (EF < 2), indicating that it was mainly from the soil. EF values of Cr, Ni, Cu, and Pb were between 10 and 100 and appeared to show significant to moderate enrichment, suggesting mixed contributions from both natural and anthropogenic sources, with more dominance from the anthropogenic activities. The EF of Cr, Cu, and Pb was notably higher (2–6 times) at the FP site compared to the FU site, likely due to the influence of nearby industrial activities (Karar et al. 2006). The proximity of the FP site to industrial areas may result in elevated emissions of Cr and Pb, primarily originating from metal processing and manufacturing industries. At the same time, Ni showed comparable enrichment at both sites. In addition to coal combustion and waste incineration, vehicle emissions, such as diesel combustion and brake lining warfare, could be sources for Cr, Cu, and Pb (Tian et al. 2012). However, the EF values of Zn, Bi, Se, and Sb were above 100, indicating the dominance of human activities such as traffic and combustion (Duan et al. 2012). Sb has the highest EF (> 1000) at both sites, as shown in Fig. 4. The high enrichment factor for Sb is attributed to local anthropogenic sources, including car brake wear, especially in densely trafficked areas (Grigoratos and Martini 2015). Further contributing to Sb’s urban presence is burning fossil fuels and incinerating Sb-containing waste (Jiang et al. 2021). Zn, Bi, and Se also have high EF (> 100), suggesting an enrichment by non-crustal sources, such as fossil hydrocarbon combustion, vehicular traffic, and emissions from metallurgical industries (Soltani et al. 2015).

### Source identification and diagnostic ratios of PAHs

**Fig. 5:**
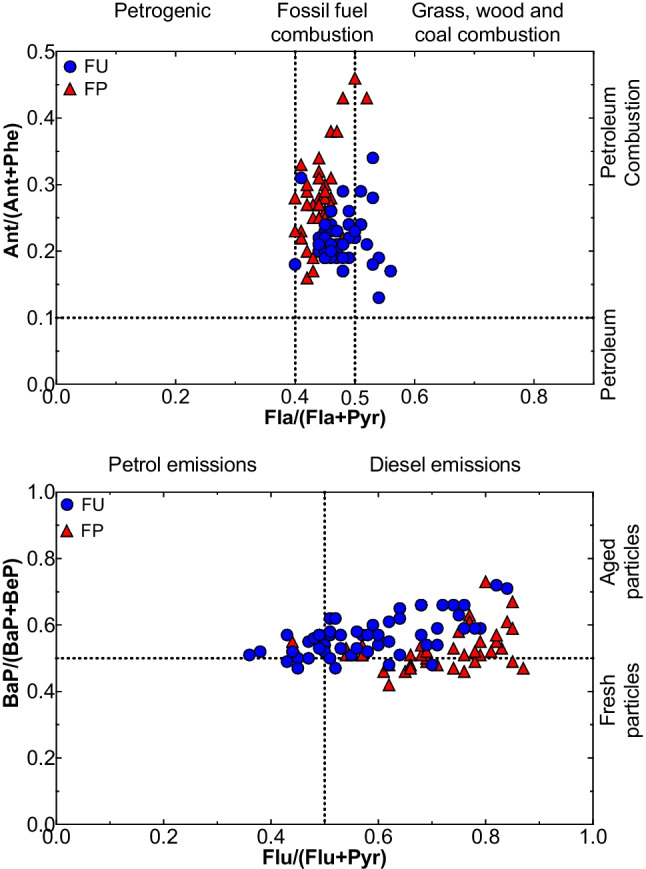
Diagnostic ratios of PAHs calculated for FU and FP sites

Many studies have applied PAH diagnostic ratios for source identification (Yunker et al. 2002; Guo et al. 2006; Sprovieri et al. 2007; Fang et al. 2007). The diagnostic ratio method for identifying PAH sources compares ratios of frequently found PAH emission pairs (Ravindra et al. 2008). Aerosol particles from traffic, domestic heating, wood combustion, and other sources are often assessed using these marker compounds (Singh et al. 2011). In addition, PAH diagnostic ratios are used to differentiate between petrol and diesel sources of fuel (Singh et al. 2011). Figure 5 provides an overview of the different PAH diagnostic ratios in this study. Ant/(Ant + Phe) ratio higher than 0.1 indicates a petroleum source, whereas Ant/(Ant + Phe) ratio lower than 1 indicates a combustion source (Pies et al. 2008; Zhang et al. 2018). The calculated ratio of Ant/(Ant + Phe) varied from 0.13 to 0.34, with a mean value of 0.22 at FU. Although the average ratio was slightly higher at FP (0.27) with a maximum value of 0.46, similar distribution patterns were observed at both sites. This suggests that the primary source of PAHs in the city is petroleum and the combustion of petroleum products.

Figure 5 shows the diagnostic ratios of PAH compounds, illustrating the different source types. The ratio Fla/(Fla + Pyr) was used to identify the origin of PAH emissions. The ratio below 0.4 indicates petroleum sources, while values between 0.4 and 0.5 are typical for liquid fossil fuel combustion sources. If the ratio is above 0.5, it suggests biomass (De La Torre-Roche et al. 2009). In this study, Fla/(Fla + Pyr) ratio was 0.40 to 0.56, suggesting that the main source is fossil fuel combustion at both sites. However, the FU site seems more influenced by biomass.

BaP/(BaP + BeP) ratios are photosensitive and often used to indicate aerosol aging and PAH photodegradation. BaP/(BaP + BeP) ratios in freshly emitted aerosols tend to be around 0.5, whereas ratios in aged aerosols might be as largely lower than 0.5 due to photochemical decomposition and oxidation (Tobiszewski and Namieśnik 2012). The BaP/BaP + BeP ratio was higher at FU (0.57) than at FP (0.53), with a majority of about 80% fresh samples at the FP site. Photochemical degradation processes play a more critical role at the FU site, with a fraction of about 54% of the total samples. In addition, the ratio of Flu/(Flu + Pyr) is also used to differentiate between diesel and gasoline PAH emissions (Ravindra et al. 2008). Most values exceed 0.5, corresponding to diesel emissions. The calculated average ratio of Flu/(Flu + Pyr) was 0.84 (FU) and 0.87 (FP), indicating the importance of diesel emissions in Fez. This shows the predominance of diesel emissions in the atmosphere, especially in the city center of Fez. However, a considerable influence of gasoline on the FU site was observed. At the same time, the BaP/BghiP ratio exceeded 0.6, which confirms the strong impact of traffic emissions on the FU site (Bortey-Sam et al. 2014). In conclusion, the diagnostic reports obtained in this study suggest that fossil fuel combustion and traffic emissions, mainly from diesel engines, are the main sources of PAHs at the studied sites in the city of Fez.

### Source apportionment of PM_10_ in Fez

**Fig. 6:**
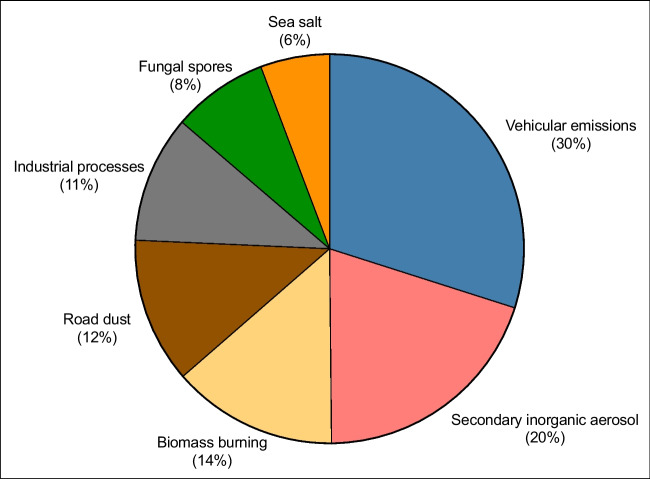
Average source contribution (in percent) for each PMF source to PM_10_ mass concentration in Fez

The average contribution of each source to the total PM_10_ concentration mass is summarized in Fig. 6. Detailed profiles of each factor are illustrated in Figure S13. It can be observed that traffic emissions contribute approximately 30% to the total PM_10_ mass. The identification of this factor is based on the substantial contribution of EC (45%), which serves as an effective tracer of direct emissions from incomplete combustion in vehicle engines, as well as the OC (42%), whose primary fraction originates from incomplete combustion. The low OC/EC ratio of the factor and the presence of traces of elements such as Cu, Sb, and Sr, which may originate from the wear of brakes, tires, and other mechanical components, as well as the contribution of organic compounds such as Ret, Flu, and Ant, emitted during the combustion of fossil fuels, corroborate this assessment. Secondary inorganic aerosols contributed 20% of the PM_10_, mostly identified by the presence of elevated contribution of SO_4_^2−^, NO_3_^−^, NH_4_^+^, and oxalate as contributors to this factor. The biomass burning factor, contributing 14% of the PM, is characterized by principal tracers such as levoglucosan, erythritol, sucrose, and coronene known to originate from biomass burning (Yang et al. 2012b; Bhattarai et al. 2019). The road dust factor explaining about 12% of the PM mass presents a complex profile of particles generated by tire wear and brake wear (Cu, Zn, Ni, Cr, Pb, Ba), pavement wear, resuspended soil and dust (Mn, K, Ti, Fe, Ca, Sr), and other materials associated with vehicle traffic and road infrastructure. This factor was assigned based on the high contribution of the crustal metals (Fe, Ti, K, Ca, Mn) and brake ware metals (Sb, Ba, Cu). The contribution from industrial processes averages 11% of the PM mass, containing a wide range of PAH compounds such as BaP, BbF, BeP, CPP, Cor, and Pyr, often linked to waste incineration, coke production, and the manufacture of iron and steel (Kong et al. 2011). Fungal spores identified by the contribution of tracers such as mannitol, sucrose, and mannose accounted for 8 of PM mass. This could be originating from trees in the forests surrounding the city of Fez. Finally, sea salt (6% of PM), primarily dominated by Cl^−^ and Na^+^ was observed, resulting from long-distance transport of air masses from coastal areas.

### Polar plots of PM_10_ sources in Fez

**Fig. 7:**
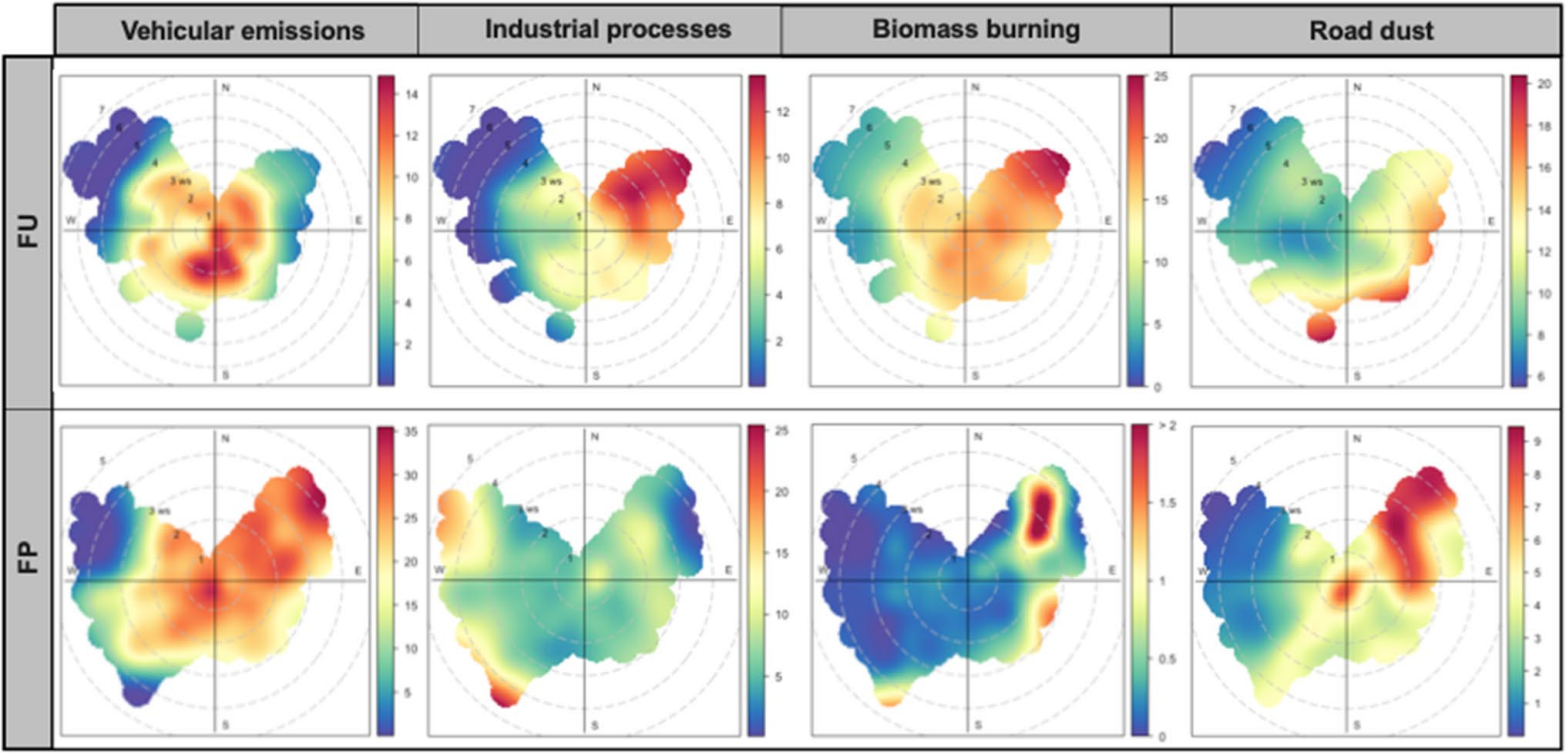
Polar plot of various PM_10_ sources, including vehicular emissions, industrial processes, biomass burning, and road dust at FU and FP sites

Figure 7 presents polar plots of anthropogenic sources, including vehicular emissions, industrial processes, biomass burning, and road dust in PM_10_ at the two Fez sites. Similar plots for sources such as biogenic fungal emissions, secondary aerosol, and sea salt are detailed in Fig. S14.

The high contribution of traffic emissions at the FU site, with a mean concentration of μg m^−3^, often originated from the south of the site during low wind conditions (< 2.5 m s^−1^), suggesting the local influence from the nearby busy traffic roads. At FP, contributions (up to 26 μg m^−3^ from local and distant traffic from the east augmented under higher wind speeds (up to 5 m s^−1^) were significant due to heavy traffic from the streets to the east of the site from light and heavy-duty trucks. Biomass burning (BB) contributed as a PM source, with a maximum attributable concentration of 5.2 μg m^−3^, at FP and was influenced by winds from the northeast and southeast, most likely from agricultural residues. At FU, a maximum BB-related PM concentration of 16 μg m^−3^ suggests local activities such as unregulated waste disposal and waste burning being important sources. Industrial emissions at FU, most probably affected by emissions from the industrial complexes El-Gaâda and Sidi Brahim, northeast to the measurement site, were observed, while at FP’s, the influence from the more westerly industrial complex Doukkarat was identified to contribute by 6.7 μg m^−3^ to PM mass.

At FP, road dust from either local or northeastern sources is a major contributor to PM air pollution. Rocade Sud, which refers to the southern ring road, produces the highest concentration of dust at 19 μg m^−3^, especially during strong eastern winds (> 4 m s^−1^). Moreover, biogenic fungal emissions peak at 9.3 μg m^−3^ at FU, while sea salt emissions peak at 11 μg m^−3^ at FP. These two sources likely come from western forests and the Atlantic coast and are transported to the sites at high wind speeds (> 4 m s^−1^). At FP, secondary aerosols are primarily produced locally due to the presumable high levels of NO_x_ and O_3_ precursors. On the other hand, at FU, aging processes during transport from the northwest and northeast play a more significant role, especially at higher wind speeds. Secondary inorganic aerosols at FU reach a maximum of 9.7 μg m^−3^ at higher wind speeds (> 3 m s^−1^). The polar plot analysis reveals that PM_10_ pollution in Fez is predominantly driven by local anthropogenic sources, with vehicular emissions, industrial activities, and biomass burning to be key contributors.

## Conclusion

The present study highlights the temporal variations of PM_10_ concentration, OC/EC, ionic species, trace metals, and PAHs in PM_10_ mass and the contribution of different PM_10_ sources using enrichment factor approaches and diagnostic ratios in Fez from September to October 2019. For the first time, a set of hydrocarbon species was measured simultaneously in aerosol samples in Morocco. Due to Saharan dust transport, the mass concentration of PM_10_ is significantly higher (107 μg m^−3^) compared to sites not affected by mineral dust. In contrast, the minimum concentration of PM_10_ (38 μg m^−3^) was recorded following precipitation events. The concentration of PM_10_ exceeded the threshold recommended by the World Health Organization (WHO) guidelines by about 81% during the sampling period. The concentrations of major PM standard compounds such as OC, EC SO_4_^2−^, NO_3_^−^, and NH_4_^+^ were comparable at both sites (Fu and FP). A significant formation of ammonium sulfate was observed through secondary particle processes in Fez, likely influenced by higher SO_2_ levels and photochemical reactions.

In contrast, the correlations between NO_3_^−^ and NH_4_^+^ were lower, possibly due to the temperature-dependent gas-particle distribution of nitrogen species. The aerosol particle standard compounds were quite similar for the two measurement sites in Fez. Considerable differences were identified with regard to PM metal and PAH content. Zn, Cu, Ni, and Pb dominated anthropogenic metals, while the most abundant PAHs were Benzo(k)fluoranthene, benzo(b)fluoranthene, and Benz(e)pyrene. Analysis of crustal enrichment factors indicated the contribution of soil dust to PM_10_ mass at both sites. However, EF for anthropogenic metals was up to 3 times higher at FP due to anthropogenic sources related to traffic and industrial tracers. The diagnostic reports clearly showed that vehicle emissions and coal combustion are the major sources of PAHs. Sucrose (192 ± 89 ng m^−3^) and mannitol (141 ± 75 ng m^−3^) were the most abundant sugar compounds at FU, while levoglucosan (27 ± 17 ng m^−3^) remained at relatively high concentrations at FP. Sucrose was strongly correlated (*r*^2^ = 0.97; *p* < 0.05) with levoglucosan and galactosan, suggesting a common source related to biomass burning emissions in Fez. Emissions from biomass combustion appear to be an important source of sugar compounds at both sites. At the same time, pollen and fungal spores play a more critical role, particularly at the FP site. Traffic emissions are identified as the primary contributor to PM_10_ concentration mass, accounting for approximately 30%, with significant roles played by secondary inorganic aerosols (20%), biomass burning (14%), road dust (12%), industrial processes (11%), and natural sources such as fungal spores and sea salt, due to their distinct chemical markers and profiles. Local anthropogenic activities, particularly vehicular emissions, industrial activities, and biomass burning, are the predominant drivers of PM_10_ pollution in Fez. As a result of combustion processes and vehicle emissions, the city of Fez may be exposed to high levels of particulate pollution, which could adversely affect its population health. According to a health risk assessment just for the PAHs studied, strategies and measures should be implemented to reduce the excess lifetime risk of cancer associated with the air quality in Fez. Further research is needed, especially on finer fractions of atmospheric aerosols, to better understand how these pollutants affect citizens’ health and to develop strategies for reducing air pollution.

### Supplementary Information

Below is the link to the electronic supplementary material.

Supplementary file1 (PDF 715 KB)

## Data Availability

The datasets used and analyzed during the current study are available from the corresponding author upon request.
