# Supplementary material for: Influence of anthropogenic activities on metals, sugars and PAHs in PM10 in the city of Fez, Morocco: Implications on air quality
Source: Environ Sci Pollut Res Int. 2024 Mar 11;31(17):25238–57. doi: 10.1007/s11356-024-32740-0 (PMC11024011; doi:10.1007/s11356-024-32740-0)
Supplement: Supplementary file 1 — Supplementary file1 (PDF 715 KB) [file 11356_2024_32740_MOESM1_ESM.pdf]

Supplementary Information (SI)

Influence of anthropogenic activities on metals, sugars and PAHs in PM_{10} in the city of Fez, Morocco: Implications on air quality

Nabil Deabji[1], Khanneh Wadinga Fomba[1], Eduardo José dos Santos Souza[1], Abdelwahid Mellouki[2,3] and Hartmut Herrmann[1*]

[1]Atmospheric Chemistry Department (ACD), Leibniz Institute for Tropospheric Research (TROPOS), Permoserstraße 15,04318, Leipzig, Germany

[2] Université Mohammed VI Polytechnique (UM6P), Lot 660 Hay Moulay Rachid, 43150, Ben Guerir, Morocco

[3]Institut de Combustion Aérothermique Réactivité et Environnement, OSUC-CNRS, 1C Avenue de la Recherche Scientifique, 45071 Orléans CEDEX 2, France

*Corresponding author: herrmann@tropos.de

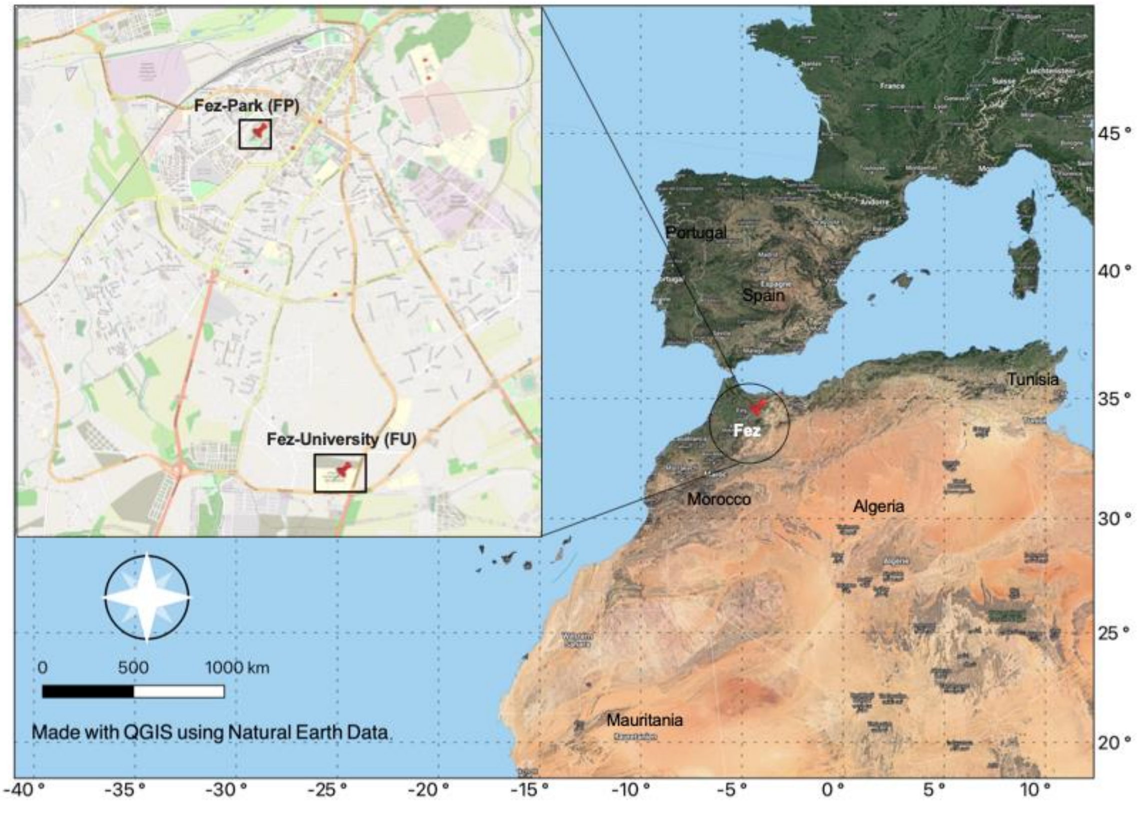

Fig. S1 Location map showing Morocco, Fez, and sampling sites.

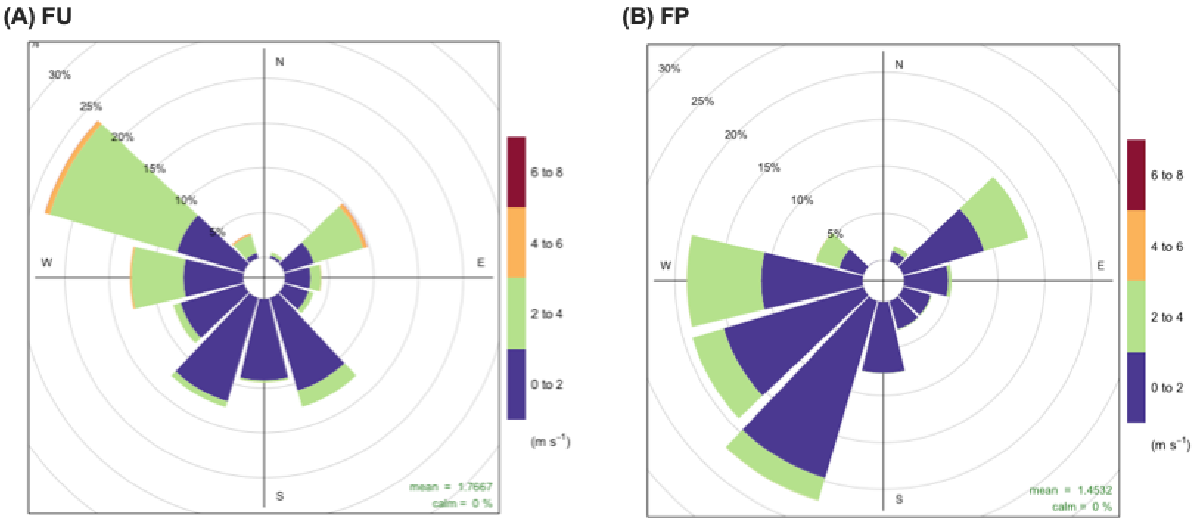

Fig. S2 Wind rose at **(A)** FU, and **(B)** FP sites. Frequency of counts by wind direction (%).

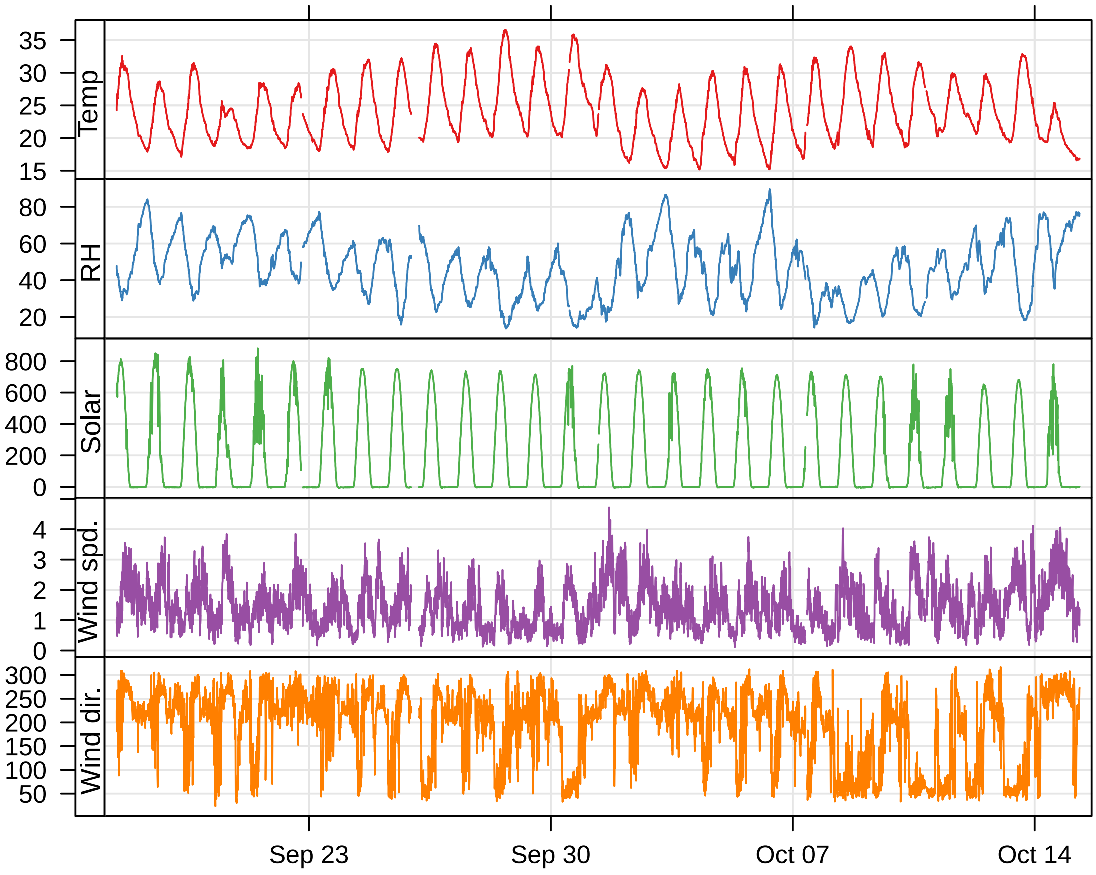

Fig. S3 Time series of meteorological parameters from 15th September to 15th October 2019 measured in Fez Parc (FP) including Temperature (Temp, °C), Relative Humidity (RH, %), Solar radiation (Solar, W/m^2), Wind Speed (Wind spd., m/s), and Wind Direction (Wind dir., degrees).

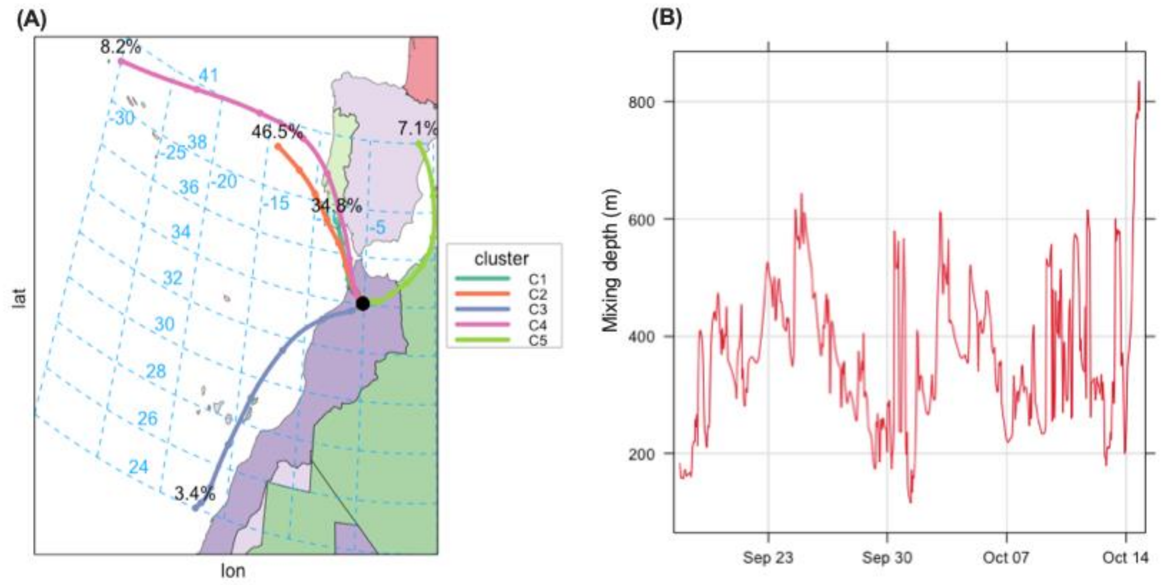

Fig. S4 (A) Air masses clusters in Fez; (B) Time series of mixing layer data.

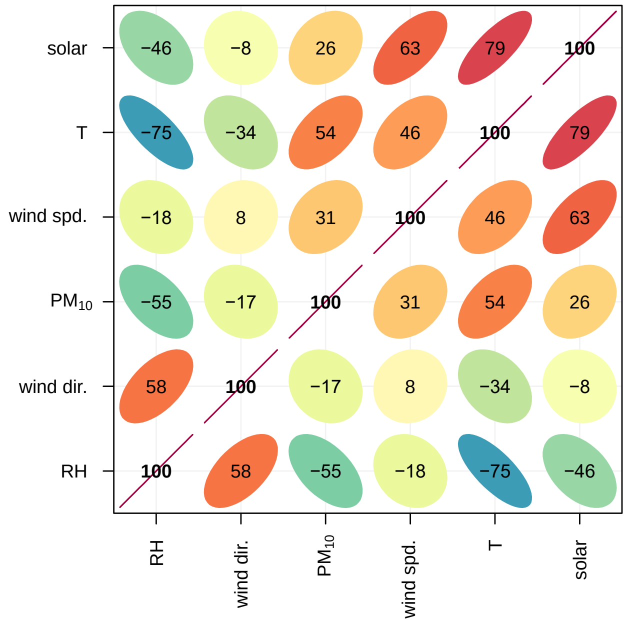

Fig. S5 Correlation matrix of PM10 mass and meteorological data (average over sampling times, measured at sampling stations). The Pearson correlation coefficient R and significance levels are show in the plot with the red color. The ellipses represent a visual representations of scatter plot with a perfect positive correlation, a line at 45 degrees positive slope.

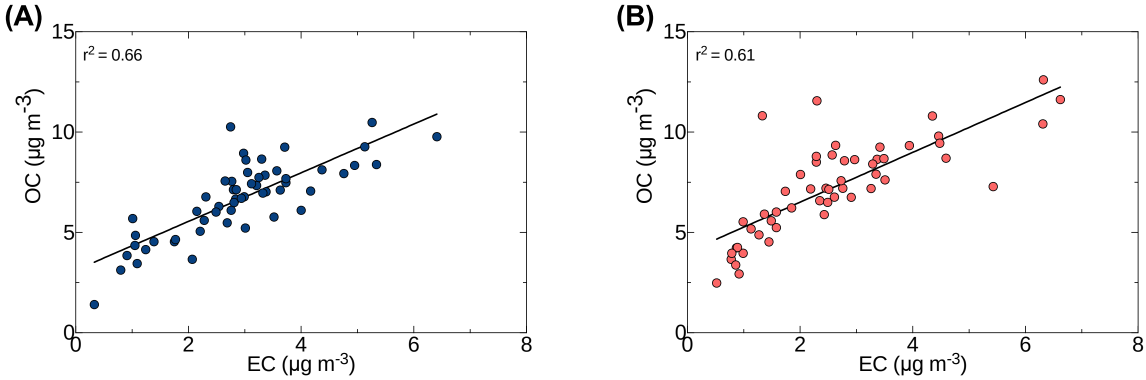

Fig. S6 Correlation plot of OC and EC at (A) FU and (B) FP.

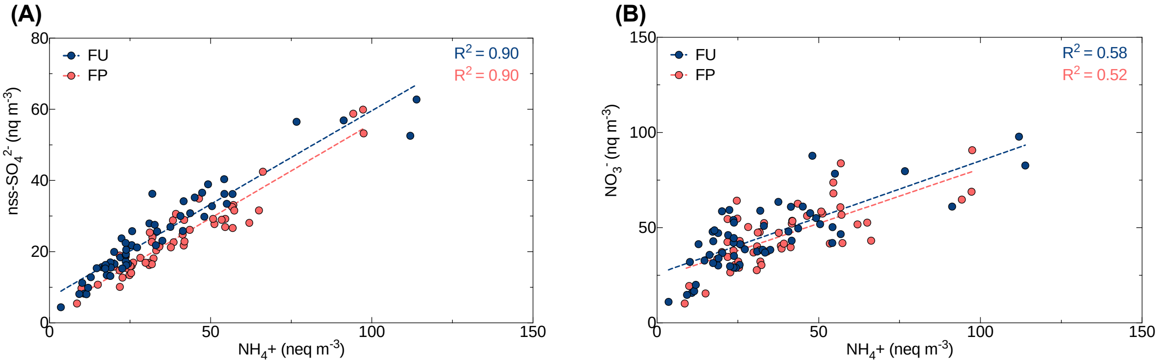

Fig. S7 Correlation plot (A)Ammonium sulfate and (B) Ammonium nitrate at each site.

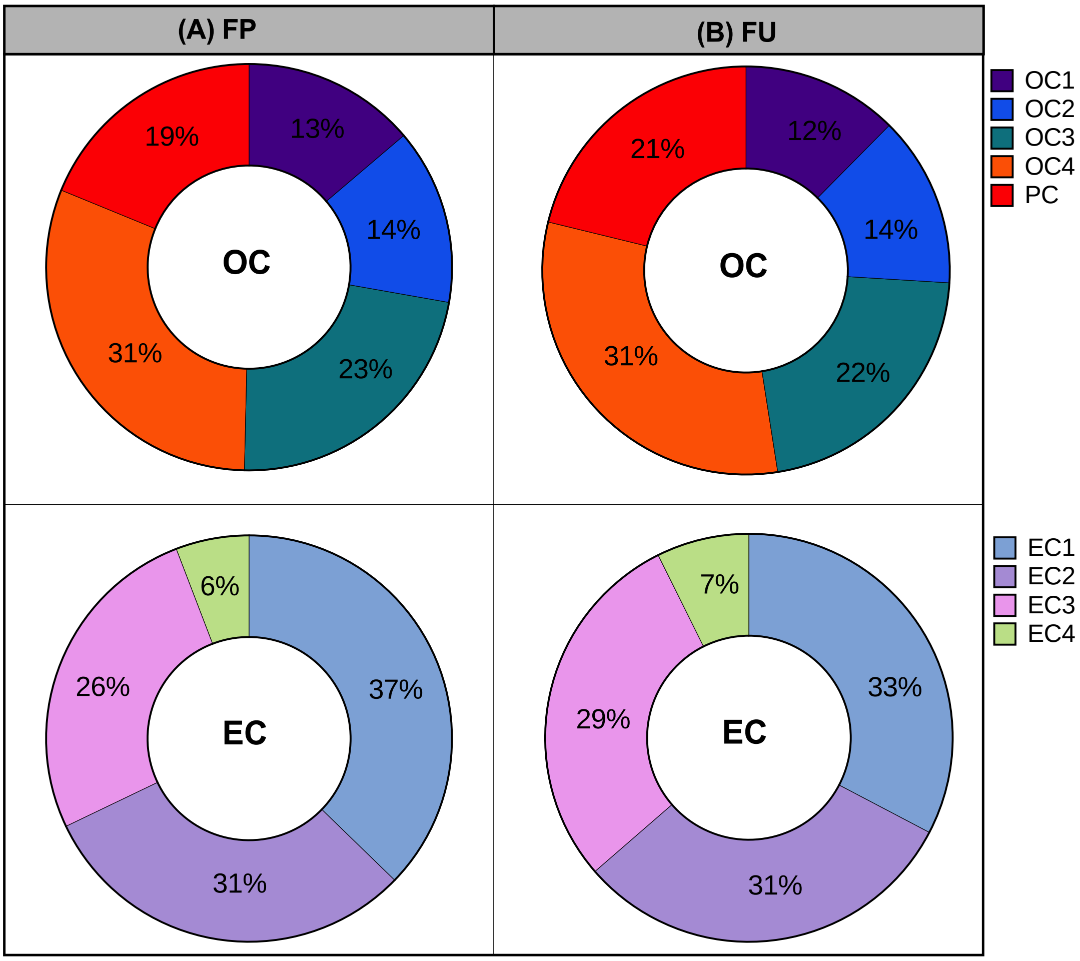

Fig. S8. Average percentage of OC and EC fraction in PM_{10} at two sites: (A) Fez University (FU) and (B) Fez Parc (FP). The diagram illustrates carbon fractions, notably organic carbons (OC_{1-4}), the organic pyrolyzed carbon fraction (PC) and elemental carbons (EC_{1-4}).

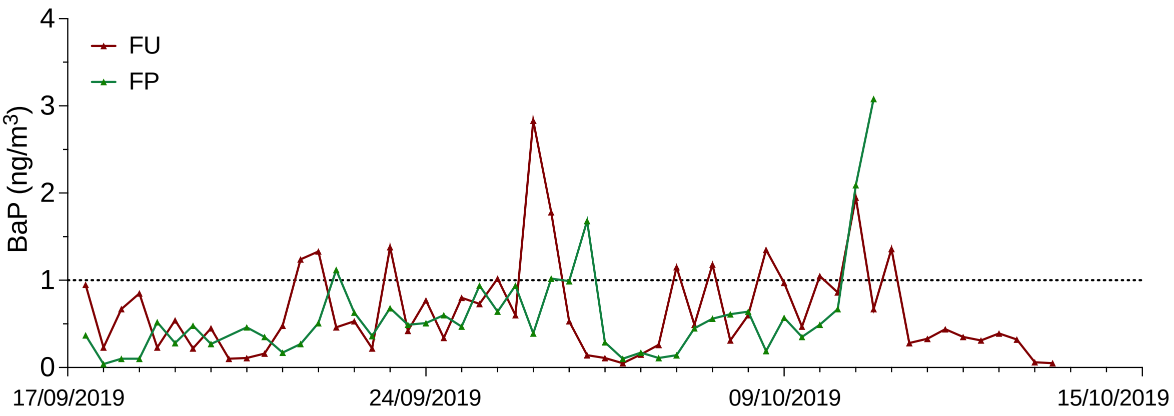

Fig. S9 Time series of BaP at both sites.

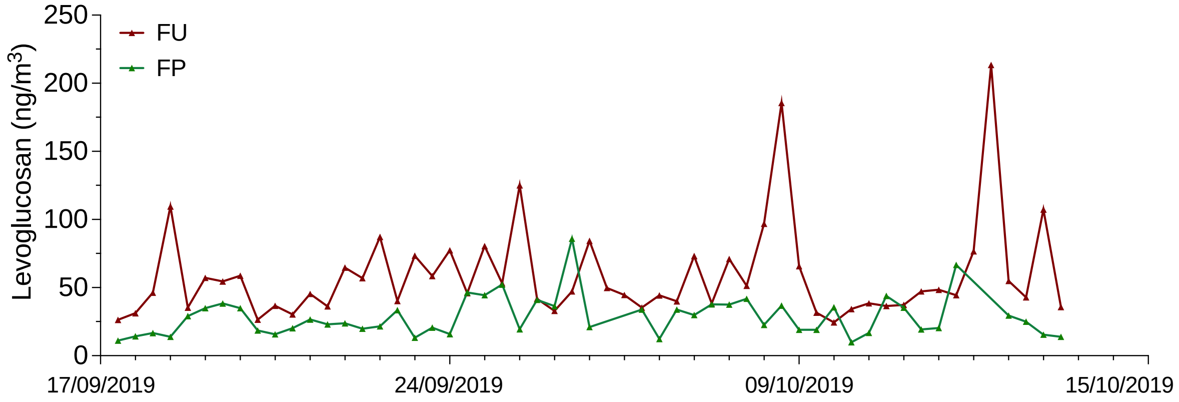

Fig. S10 Time series of levoglucosan at both sites.

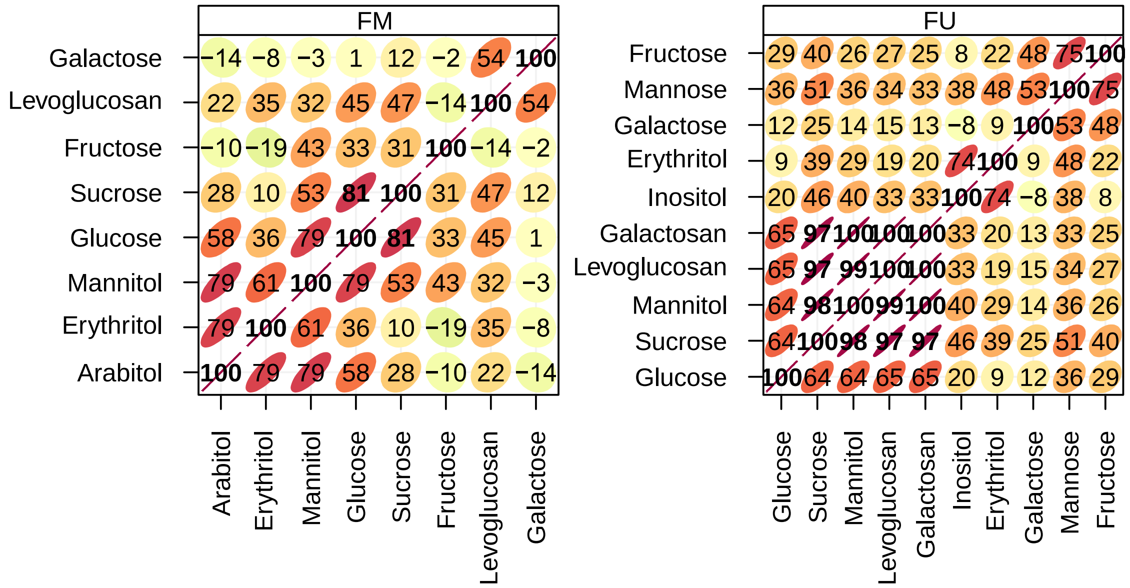

Fig. S11 Correlation plot of sugar compounds at each site.

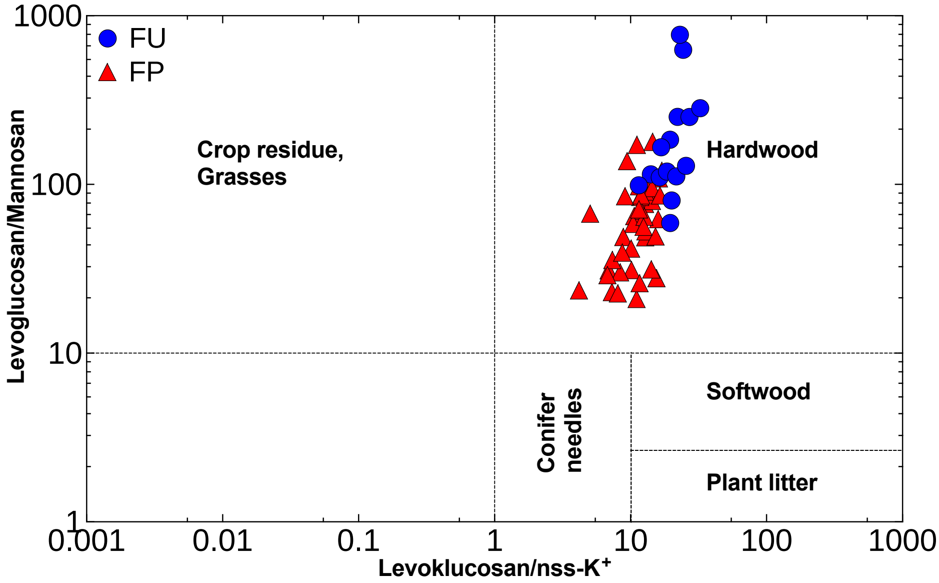

Fig. S12 Scatter plot of Levoglucosan/nss-K+ versus Levoglucosan/Mannosan ratios in PM_{10} collected in Fez.

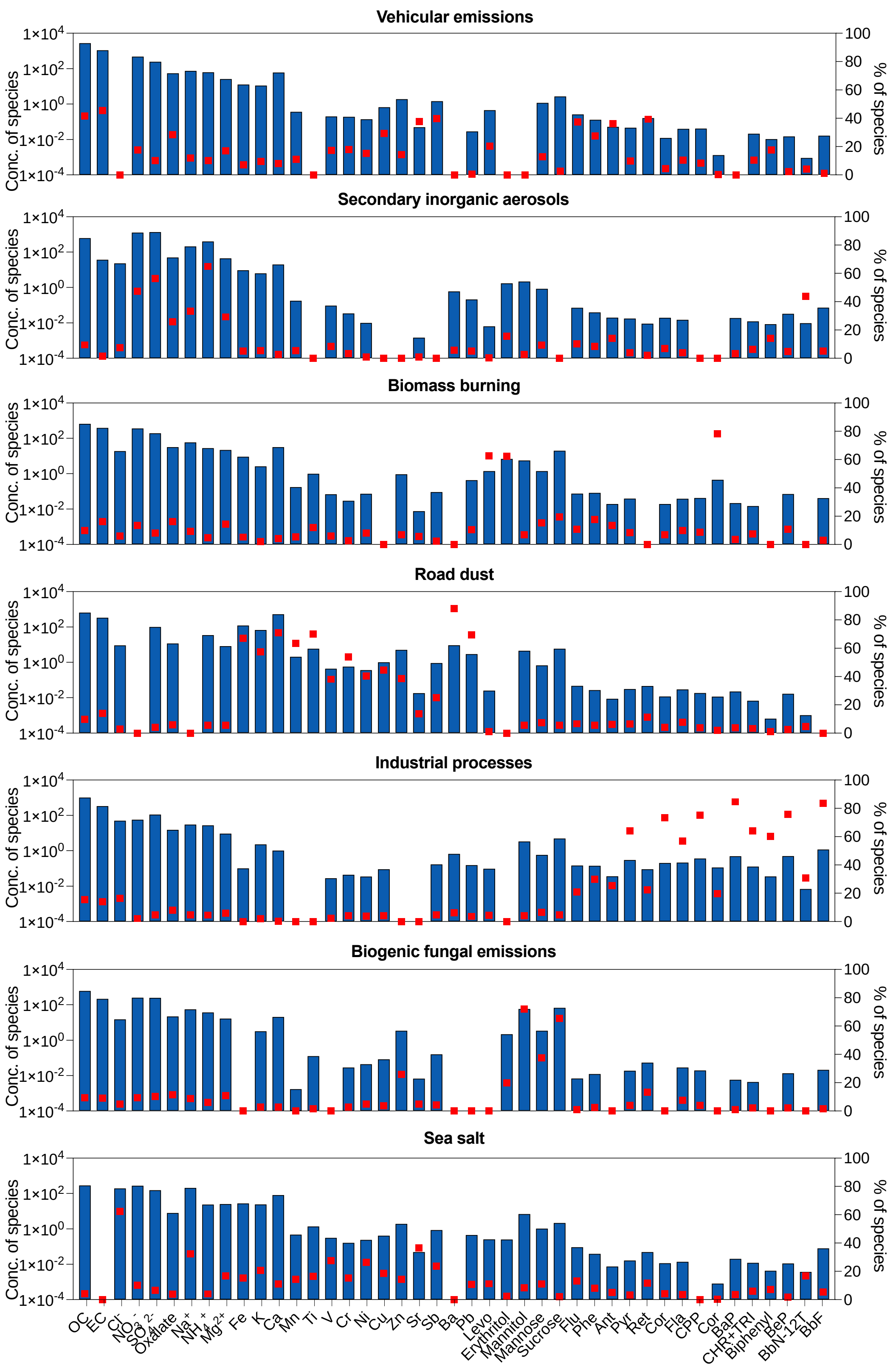

Fig. S13 Overview of PMF source profiles at FEZ sites, including species concentrations (left axis) and the percentage of species associated with each source (right). PMF profile species concentrations (ng m^{-3}) are shown in blue bars, and red dots indicate the contribution (%) of the chemical species associated with the source.

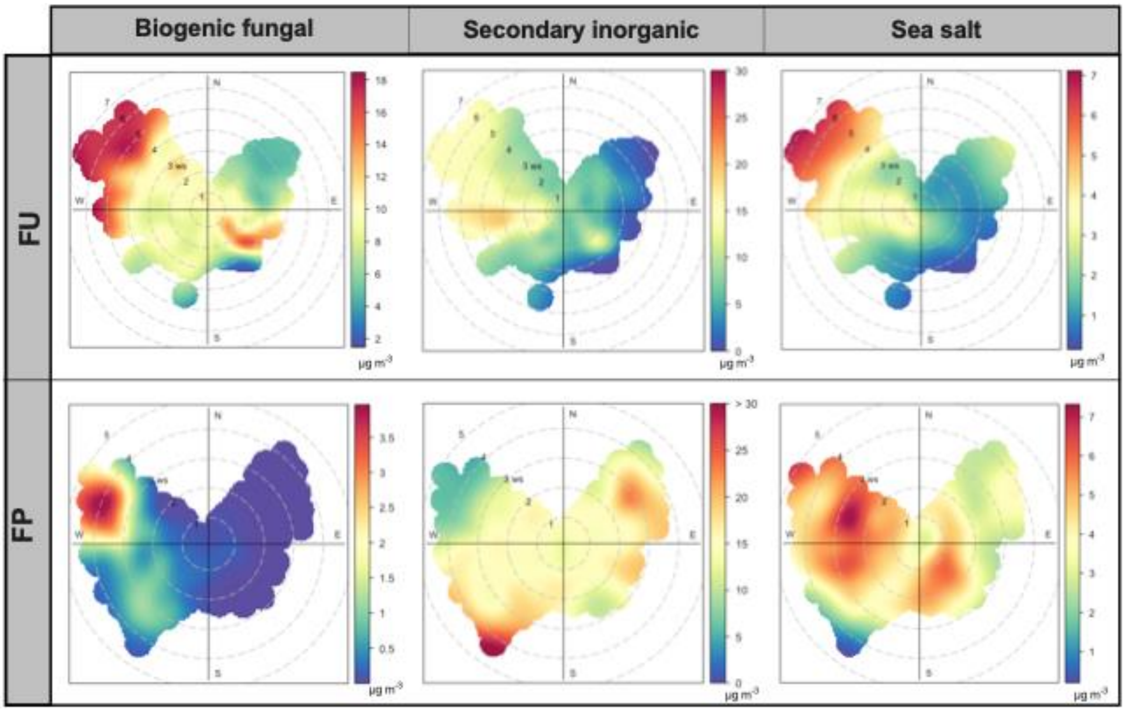

Fig. S14 Polar plot of various PM_{10} sources, including biogenic fungal, secondary inorganic aerosol, and sea salt at FU and FP sites.

Tab. S1 List of organic compounds and their respective symbols measured in aerosol particle samples in Fez.

Compounds	Symbol
Fluorene	Fla
Phenanthrene	Phe
Anthracene	Ant
Pyrene	Pyr
Retene	Ret
Coronene	Cor
Fluoranthene	Fla
Cyclopenta(cd)pyrene	Phe
Benz(a)anthracene	BaA
Benzo(k)fluoranthene	BkF
Benz(a)pyrene	BaP
Chrysene	Chr
Chrysene(+Triphenylene)	Chr/Tr
Benzo(ghi)perylene	BghiP
Dibenzo(ah)anthracene	DahA
Benz(e)pyrene	BeP
Benzo(b)fluoranthene	BbF
Indeno(1 2 3-cd)pyrene	IcdP

Tab. S2 Concentration of identified of sugar compounds in the aerosol particle samples at FU and FP sites.

Sugar	FU		FP	
Compound	Mean	SD	Mean	SD
Anhydrosugars	119	73	28	17
Levoglucosan (Lev)	58	35	27	15
Mannosan (Man)	0.91	1.7	2.2	1.6
Galactosan (Gal)	60	36.5	-	
Monosaccharides	221	89	22	14
Mannose	12	3.4	-	
Glucose	0.27	0.38	9.8	3.9
Galactose	0.27	4.3	0.04	0.29
Fructose	14	7.3	0.62	4.5
Sucrose	192	89	12	8.8
Sugar alcohols	164	77	24	12
Arabitol	-	-	9.9	4.6
Inositol	2.9	1.2	-	-
Erythritol	19	6.7	1.5	1.1
Mannitol	141	75	14	6.5
